# Enhancing cassava N/K use efficiency through Fenlong tillage: driving forces of soil porosity improvement and aerobic bacteria enrichment

**DOI:** 10.3389/fmicb.2026.1739897

**Published:** 2026-02-11

**Authors:** Guilong Li, Xiaohui Peng, Li Gan, Yuanhang Huang, Fengyan Qin, Liangwu Li, Jia Zhou, Weixian Yang, Zhangyou Shen, Maogui Wei

**Affiliations:** 1 College of Agronomy, Guangxi University, Nanning, China; 2 Cash Crops Research Institute, Guangxi Academy of Agricultural Sciences, Nanning, China; 3 Guangxi Key Laboratory of Agro-environment and Agro-products Safety, Guangxi University, Nanning, Guangxi, China

**Keywords:** aerobic bacteria, cassava, Fenlong tillage, fertilizer use efficiency, soil microbial community

## Abstract

Cassava (*Manihot esculenta* Crantz) is continuously cultivated using the conventional tillage (CT) method in southern China, resulting in increasingly compact soil and decreased yield and fertilizer use efficiency (FUE) year after year. Compared to CT, Fenlong (FL) tillage, which uses spiral drill bits to replace traditional ploughshares, has been found to significantly increase crop yield ranged from 10% to 50% such as cassava, sugarcane, maize, cotton, rice, and wheat without extra fertilizer input. However, previous studies have primarily elucidated the mechanism behind yield increases from FL by examining changes in soil physicochemical properties and microbial communities. Research on how it enhances FUE remains scarce. Thus, the current study aimed to investigate how FL influences both cassava yield and FUE, which would be of great significance for implementing the plan “increasing crop yield without increasing fertilizer” released by the Ministry of Agriculture of China. Results indicated that the soil porosity of the FL treatment was 2.07 to 38.37% higher than that of the CT group. Moreover, compared with CT, the soil bulk density under FL treatment decreased by 0.64% to 12.07% across different soil layers, with significant reductions observed in the 11 ~ 30 cm layers in 2019 and the 21 ~ 30 cm layer in 2020. The relative abundance of aerobic bacteria (i.e., Gemmatimonadaceae [Family] and *Tumebacillus [Genus]* in 2019; Micromonosporaceae [Family], *Tumebacillus [Genus]*, *Conexibacter [Genus]*, and Acidobacteriales [Order] in 2020) in the FL group was higher than that in CT. The FL treatment significantly outperformed CT, with increases ranging from 9.04 to 135.81% in tuberous root yield, 11.51 to 62.07% in FUE-N, and 21.75 to 40.76% in FUE-K, while the fertilization regime of N 118.2 kg.ha^−1^, P_2_O_5_ 29.4 kg.ha^−1^, and K_2_O 61.9 kg.ha^−1^ under FL managed to reach an ideal balance between cassava yield and FUE. Structural equation modeling revealed that FL tillage improves soil conditions for cassava and aerobic bacteria through deep soil fragmentation. This promotes soil fertility, facilitates deeper root penetration for enhanced nutrient and water uptake, and ultimately leads to higher yield and FUE compared to CT.

## 1 Introduction

Cassava (*Manihot esculenta* Crantz) is widely cultivated in tropical and subtropical areas as one of the most important food crops for nearly one billion people in developing countries. In China, cassava is mainly cultivated in the tropical and subtropical regions, primarily in the following provinces: Guangxi, Guangdong, Hainan, Fujian, Yunnan, Jiangxi, and Guizhou. Among these, Guangxi is the largest cassava-producing region in China, accounting for over 60% of the country’s total planting area and yield. As a C3 plant with high photosynthetic efficiency, cassava has a theoretical yield potential of up to 120 t/ha (Howeler et al., 2013), while high-yield varieties also achieved a yield record of 75 t/ha in China (Liang et al., 2017). Cassava is mainly cultivated on marginal land with slopes, which suffer from soil erosion, leading to far more severe nutrient losses than from flat fields (Wang et al., 2018), thus resulting in an average yield that was 4–5 times lower than the high-yield record (16.43 t/ha, Food and Agriculture Organization, 2025). In addition, continuous cassava cropping is prevalent, largely due to limited land resources and traditional farming practices (Chen et al., 2025; Zhu et al., 2025). With conventional tillage (CT) method applied over years, the plow layer has become shallow and compacted, particularly in the poor soils of southern China’s hilly regions. This has resulted in declining fertilizer use efficiency (FUE) and a year-by-year decrease in field productivity (Yu et al., 2022; Liu et al., 2020; Zhang et al., 2012). Thus, agricultural management practices must be further developed for sustainable cassava production, e.g., applying new tillage methods and using fertilizers more efficiently.

As a field preparation measure, tillage can directly change the bulk density, porosity, and particle size distribution of the soil, and indirectly affect its chemical properties and microbial communities, ultimately influencing crop yields (Ahmadi et al., 2024; Adeleke et al., 2023; Ferreira et al., 2023; Pečan et al., 2023; Amami et al., 2021; Chichongue et al., 2020; Woźniak, 2019). The intensity and the mode of mechanical disturbance on soil varied resulting in different tillage methods, ending up with different crop growth traits. The CT method for dry-land preparation in China is mouldboard ploughing once at 30 ~ 35 cm depth and raking the topsoil twice (depth≈18 cm). Previous studies declared that when the CT method was applied on the same field for years, soil physical and chemical properties severely degrade with decreasing soil aggregate stability and organic matter content (Ahmadi et al., 2024; Thomaz and Antoneli, 2022; Bueno and Ladha, 2009), ending up with a decline of crop yield and FUE year after year (Kumari et al., 2023; Schlegel et al., 2018; Habbib et al., 2017). To maintain crop yield under the CT treatment, the input of chemical fertilizers increased year by year, especially nitrogen, which is an essential element for crop development.

However, excessive chemical fertilizer application not only reduces the FUE of crops by reducing root mass but also causes numerous adverse effects on soil and the environment (Ordóñez et al., 2021; Varvel and Peterson, 1990). Toxic substances from inorganic fertilizers accumulate in the soil when massive fertilizers are applied, leading to the decline of soil quality and the reduction of soil microorganisms (Sun et al., 2012). Furthermore, nitrogen loss from the field had been proven as the major source that causes agricultural non-point source pollution, threatening the environment and human health (Yan et al., 2023; Bowles et al., 2018; Liu et al., 2010; Gruber and Galloway, 2008; Galloway et al., 2003). Improving crop FUE has become one of the most important challenges in modern agriculture. Although reducing nitrogen fertilizer application can enhance the FUE of N, crop development would be constricted suffering by N deficiency, resulting in low biomass (Liu et al., 2024; Xu et al., 2018). Nevertheless, nitrogen use efficiency can be improved with a satisfactory crop yield by integrating tillage treatments, irrigation practices, and biochar use with fertilization (He et al., 2024; Xiao et al., 2023; Shah et al., 2016). Thus, to improve FUE, integrated fertilizer management measures in parallel with other agronomic approaches are needed for sustainable crop production.

As a new tillage method, Fenlong tillage (FL), has been referred to as deep vertical rotary tillage (Li et al., 2020; Zhai et al., 2017, 2019), smash ridge tillage (Bai et al., 2021; Zhang et al., 2021), or Fenlong ridging (Duan et al., 2022) in previous studies, uses spiral drill bits replacing traditional ploughshares, which can fully break and level the soil with depths up to 30 ~ 60 cm at once. Compared to no tillage and subsoil tillage, FL significantly increased the root dried matter and grain yields of summer maize (*Zea mays* L.) and nitrogen use efficiency by reducing the soil bulk density of the 0–40 cm layer (Zhai et al., 2017, 2019). Compared to CT, FL was applied on rice (*Oryza sativa* L.) and significantly improved root morphology and physiology by optimising the soil physical properties and increasing contents of organic matter (OM), available phosphorus(AP), alkaline-hydrolyzable nitrogen (AN), available potassium (AK), and soil oxidation–reduction potential, ending up with a higher grain yield (Zhang et al., 2021). When it was applied to sugarcane (*Saccharum officinarum* L.), the growth-promoting effects of FL on sugarcane were also observed, and FL was also found to decrease soil bulk density, increase soil water storage ability to some extent, promote the activity of endophytic microbes in the roots, resulting in a well-developed root system and satisfied sugarcane yield (Duan et al., 2022; Li et al., 2020). Compared with CT, FL has been reported to increase crop yield by 10 to 50% with less fertilizers input (Geng et al., 2022; Li et al., 2020). Furthermore, FL has also been shown to significantly improve nitrogen use efficiency in maize and potassium use efficiency in potatoes (*Solanum tuberosum* L.) (Geng et al., 2022; Zhai et al., 2019). Thus, to assess the potential of FL for enhancing fertilizer utilization in cassava and to uncover its underlying mechanisms, the combined effects of FL and fertilizer applications on cassava were evaluated in the current study. CT was set as the control, while eight fertilizer application rates were investigated. The study examined both the promoting effects of FL on soil-cassava systems and the structure and diversity of the dominant bacterial and fungal communities across treatments.

## 2 Materials and methods

### 2.1 Experiment site

**Figure 1:**
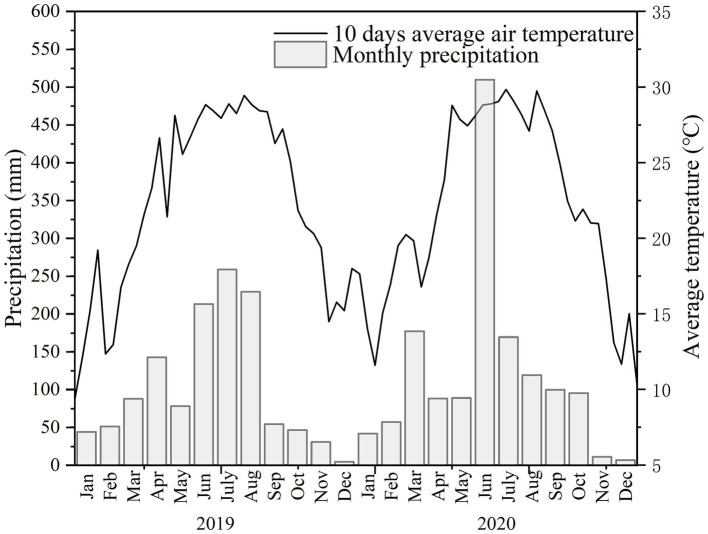
Weather data during the 2019~2020. The figure shows monthly precipitation (mm) and ten-day average air temperature (°C).

The experiment was conducted in an experimental base within Wuming County (22°59′58″N, 107°49′26″E), Guangxi, China, in 2019 and 2020. The climate there is a subtropical monsoon climate, with an average annual precipitation of 1,233 mm and an average annual temperature of 21.7 °C (Figure 1). The field was left fallow for 2 years before the experiment was conducted, and its soil physical and chemical characteristics were well described in Li et al. (2023).

### 2.2 Experiment design and operation

A split plot design was adopted for the field experiment (Supplementary Figure S2). Tillage treatment was designated as the main factor, and fertilization regimes were set as the secondary factor. Two tillage methods, CT and FL, were applied, and two blocks were first split along the long dimension of the experimental field for CT and FL, respectively. To ensure stable and high-yielding cassava production with improved fertilizer use efficiency, a balanced fertilization experiment was also included. The recommended NPK application rate for cassava, as proposed by local research institutions, was N 358.80 kg·ha^−1^, P_2_O_5_ 89.10 kg·ha^−1^, and K_2_O 187.50 kg·ha^−1^ (Gao et al., 2011), representing a nutrient ratio of approximately N: P_2_O_5_: K_2_O ≈ 4: 1: 2. This recommended rate was defined as the 100% NPK treatment (100 NPK). Based on this benchmark, three reduced fertilization levels were established: 33% NPK (33NPK), 66% NPK (66NPK), and a zero-fertilizer control (0 N0P0K, CK). To address the negative consequences of nitrogen over-application on the environment and soil, reduced-nitrogen treatments were incorporated into the experimental design. In total, eight fertilization rates were then set, replicated three times, and randomly arranged within the tillage treatment blocks, resulting in a total of 48 plots (Table 1). The individual plot size was 14 m × 5 m, ending up with 70 plants in each plot with a row spacing of 1 m × 1 m.

**Table 1:** Design of the fertilization regimes.

Experiment		Fertilizer rate(kg·hm^−2^)		
		*N*	P_2_O_5_	K_2_O
CK	N_0_P_0_K_0_	0	0	0
0N	N_0_P_100_K_100_	0	89.1	187.5
25N	N_25_P_100_K_100_	89.5	89.1	187.5
50N	N_50_P_100_K_100_	179.0	89.1	187.5
75N	N_75_P_100_K_100_	268.6	89.1	187.5
100N	N_100_P_100_K_100_	358.8	89.1	187.5
33NPK	N_33_P_33_K_33_	118.2	29.4	61.9
66NPK	N_66_P_66_K_66_	236.3	58.8	123.8

Cassava variety South China 205 was used as plant material, which was one of the most popular cultivated varieties in China. Tillage was implemented for field preparation on April 15, 2019. Given the high soil disturbance associated with FL tillage, it was commonly practiced on an alternate-year basis. Consequently, on April 9, 2020, all plots underwent only two passes of raking to a depth of 18 cm. Cassava was sown on the date after field preparation and harvested on January 13th 2020 and January 17th 2021, respectively. No base fertilizer was used, and the designed fertilizers were applied as topdressing, 60% were applied on the 60 days of planting (DAP) and the rest were applied on the 120 DAP. No artificial watering was added. The planting method, fertilization amount and field management in 2020 were all consistent with those in 2019.

### 2.3 Assessment of cassava agricultural traits, yield and FUE

Five plants were randomly selected from each plot. The plant height and the stem diameter (the diameter of the cassava stem at about 15 cm above the ground) of these plants were tracked and measured. The plant material sampling was conducted during the cassava root formation period (July 14), expansion period (September 18), maturity period (December 15), and harvest time (January 13, 2020) in 2019, while the samples for 2020 were collected on July 11, September 17, December 18, 2020, and January 17, 2021, respectively. The fresh weight (FW) of cassava tuberous roots and stems were measured and transported to the laboratory, cleaned, chopped into small fractions (<2cm^3^), and thoroughly homogenized. Quartering method was then applied, and about 500 g of fresh sample was taken for each plot, oven-dried at 105 °C for 15 min, dried to constant weight at 60 °C, ground to pass through an 80-mesh sieve, and then preserved in plastic bags before wet chemical analysis. The root and stem yield of cassava were calculated using the same formula: (1) *yield (kg.hm^−2^) = 10,000 (plant population/hm^2^) × FW (kg)*. To evaluate the leaf yield, 10 cassava plants were randomly selected for each plot. The average leaf number of each plant (ALN) was estimated by calculating the remain green leaves and the leaf scars. Ten mature green leaves were taken and oven-dried to calculate the average weight of a single leaf (ASL). Leaf dry matter yield (LDY) was calculated using formula (2) *LDY (kg.hm^−2^) = 10,000 (plant population/hm^2^) × ALN× ASL (kg).*

Plant samples were digested using H_2_SO_4_-H_2_O_2_ method (Bao, 2000). Contents of nitrogen and phosphorus were then determined by using the automatic intermittent chemical analyser (SmartChem 200, Zeal Quest Equipments), while potassium content was measured using a flame photometer (WGH6400, Shanghai Changxi Instrument & meters Co., Ltd.). The nutrient accumulation (NA) of N, P, and K of each plot was calculated by using formula (3) *NA (kg.hm^−2^) = root and stem dry-matter yield (kg.hm^−2^) × nutrient content (%).* The FUE of each element was then calculated accord to formula (4) (Fageria and Baligar, 2003):


$$\mathrm{FUE}(\%)=\frac{N_{f}-N_{u}}{N_{a}}\times 100\;$$

where *N_f_* and *N_u_* are the nutrient accumulation of fertilized plots (kg) and unfertilized plots (kg), respectively, while *N_a_* is the quantity of nutrient applied (kg).

A comprehensive evaluation of each treatment in terms of cassava yield and FUE was conducted using principal component analysis (PCA). The model included only principal components with eigenvalues greater than 1. The composite score for all fertilizer treatments under FL tillage was calculated using the equation as follows (Zhang et al., 2023):


$$f=\sum (0.46196\times \frac{T1_{j}}{\sqrt{2.772}}+0.23947\times \frac{T2_{j}}{\sqrt{1.437}})\times X_{j}$$

*J* = 1,2,3,4,5,6.

In the equation, T1*_j_* and T2*_j_* represent the scores of each variable on principal component 1 and 2, respectively, derived from the PCA component score matrix. X*_j_* denotes the original measured value of the yield of root and stem, and FUE in 2019 to2020. The coefficients 0.46196 and 0.23947 are the variance percentages explained by component 1 and component 2, respectively, which correspond to their initial eigenvalues of 2.772 and 1.437.

### 2.4 Soil sampling and analysis

To evaluate how tillage treatments influence the soil bulk density and porosity, five sampling points of each tillage treatment were randomly chosen according to the S-shaped route (Xiao et al., 2020) and the samples of soil profile with three layers of 0 ~ 10 cm, 11 ~ 20 cm, and 21 ~ 30 cm of each point were collected before planting and harvesting in 2019 and 2020, respectively. Soil samples for all layers were collected by ring knives, marked, brought back to the laboratory, and measured (Li et al., 2022).

To measure the chemical properties, soil samples were collected on July 14 (the cassava root formation stage), September 18 (root expansion stage), and December 15 (root maturity stage) in 2019 and 2020, respectively. Five sampling points were randomly selected with an S-shaped pattern within each plot. The impurities on the soil surface were removed before sampling. Soils from the 0 ~ 20 cm depth at these five points were collected using a soil sampler and thoroughly mixed. The quartering method was subsequently applied to obtain a representative soil sample of approximately 500 g per plot, and then air-dried. To measure the soil microorganisms, soil samples collected during the root maturity stage of 0 N, 25 N, 50 N, and 100 N treatments were immediately divided into two portions after mixing and quartering. One portion (100 g) was immediately frozen with liquid nitrogen and stored at −80 °C for the determination of soil microorganisms, while the other part was air-dried for soil chemical properties detection.

The pH value was detected using a PHS-2F meter (Shanghai Inesa Scientific Instrument Co., Ltd.) with a soil-water ratio of 1: 2.5 (W/V). The content of organic matter (OM) was measured by the potassium dichromate volumetric method. Alkali-hydrolysable nitrogen (AN) was measured by alkaline hydrolysis diffusion method, while available phosphorus (AP) was measured by molybdenum acid colorimetry, and available potassium (AK) was measured by NH_4_OAc extraction and a flame photometer (WGH6400, Shanghai Changxi Instrument & Meters Co., Ltd.). Details of these methods were well described by Bao (2000).

### 2.5 Soil microbial analyses

The DNA of bacteria and fungi in soil samples was extracted using the FastDNA® Spin Kit for Soil (MP Biomedicals, USA) and the E.Z.N.A.® Soil DNA Kit (Omega Bio-Tek, Norcross, GA, USA) according to the manufacturer’s instructions, respectively. For bacteria, the V3-V4 hypervariable regions of the 16S rRNA gene were targeted using primers 338F (5’-ACTCCTACGGGAGGCAGCAG-3′) and 806R (5′-GGACTACHVGGGTWTCTAAT-3′), while primers SSU0817F (5′-TTAGCATGGAATAATRRAATAGGA-3′) and 1196R (5′-TCTGGACCTGGTGAGTTTCC-3′) were used to amplify the target fragment of the 18S rRNA sequence in the SSU0817F_1196R region for fungi (Borneman and Hartin, 2000). The DNA was amplified by PCR according to the method described Li et al. (2023). The PCR products were separated using a 2% agarose gel, then purified and quantified using the AxyPrep DNA Gel Extraction Kit (Axygen Biosciences, Union City, CA, USA) and the Quantus™ Fluorometer (Promega, USA), respectively. Library was constructed using the NEXTFLEX Rapid DNA-Seq Kit. Paired-end sequencing (2 × 300 bp) was conducted using the Illumina MiSeq platform (Illumina, San Diego, CA, USA) by Majorbio Bio-Pharm Technology Co., Ltd. (Shanghai, China). All raw reads of bacteria and fungi obtained in this study have been deposited in the National Center for Biotechnology Information (NCBI) with accession numbers PRJNA1188335 and PRJNA1188458, respectively.

Quality filtering and adapter trimming of the demultiplexed reads were performed using Trimmomatic software. Flash software was used to filter and splice the data with the following criteria: (1) reads were trimmed when the average quality score fell below 20 within a 50-bp sliding window; (2) sequences were merged if they overlapped by more than 10 bp with no more than 2 mismatches; (3) sequences of each sample were separated according to barcodes (exactly matching) and primers (allowing two nucleotide mismatching), and reads containing ambiguous bases were removed. Subsequently, the Upaste algorithm (Upaste v7.1) was used to cluster the validated tags across all samples, with sequences being grouped into operational taxonomic units (OTUs) under the standard 97% similarity criterion. The RDP classifier 2.11 was used to annotate the screened non-chimeric sequences for species classification, using a threshold of 0.7 against the Silva database (SSU138 for fungi, Release_138 for bacteria, http://www.arb-silva.de/). Alpha diversity analysis (including the Shannon, Simpson, Ace, and Chao indexes) was carried out using MOTHUR software. In addition, the *R 3.3.1* was used for data mining and plotting.

### 2.6 Statistical analysis

Statistical analysis, such as analysis of variance (ANOVA), paired *t*-test, multiple comparison with the Tukey HSD, and PCA modeling was performed using IBM SPSS Statistics 20.0, while plotting was done using OriginPro 2022. The structural equation model was generated by the SPSSPRO online platform (https://www.spsspro.com/analysis/index, accessed on 15 August 2025).

## 3 Results

### 3.1 Effect of tillage treatment and fertilization regime on soil physical and chemical properties

Multiple comparison of soil physical properties revealed that there were differences in soil bulk density and porosity in all layers between the two tillage treatments in 2019 after the first cassava planting season (Table 2). Soil bulk density of the 11 ~ 30 cm layers (1.37 ~ 1.53 g‧cm^−3^) in 2019 and the 21 ~ 30 cm (1.67 g‧cm^−3^) layer in 2020 under the FL treatment were significantly lower than that of the CT group (1.50 ~ 1.74 g‧cm^−3^ of 11 ~ 30 cm and 1.81 g‧cm^−3^ of 21 ~ 30 cm), while the difference gap of the 21 ~ 30 cm layer between two tillage treatments became narrower after the secon cassava planting season (2019, 1.53 g·cm^−3^ of FL, 1.74 g·cm^−3^ of CT; 2020, 1.67 g·cm^−3^ of FL, 1.81 g·cm^−3^ of CT). Soil porosity of the FL treatment (2019, 45.25% ~ 47.35%; 2020, 32.12% ~ 43.94%) was generally higher than that of the CT group (2019, 34.22% ~ 43.53%; 2020, 31.47% ~ 39.68%). In addition, soil chemical properties were influenced by both tillage and fertilizer treatments. Notably, tillage showed stronger influences on the nutrient parameters than the fertilizer treatment according to the mean square of ANOVA results, especially in 2019 (Table 3, Supplementary Figure S3).

**Table 2:** Effects of tillage on soil bulk density and porosity.

Tillage	Depth (cm)	Bulk density (g‧cm^−3^)			Soil porosity (%)		
		Before planting	Before harvest in 2019	Before harvest in 2020	Before planting	Before harvest in 2019	Before harvest in 2020
FL	0 ~ 10	1.06 ± 0.03b	1.40 ± 0.23bc	1.55 ± 0.11b	59.15 ± 1.22ab	45.73 ± 4.54ab	43.94 ± 3.44a
	11 ~ 20	1.02 ± 0.11b	1.37 ± 0.01c	1.61 ± 0.05b	59.24 ± 4.02ab	45.25 ± 1.80ab	40.97 ± 1.33a
	21 ~ 30	1.11 ± 0.06ab	1.53 ± 0.08b	1.67 ± 0.02b	58.17 ± 2.15b	47.35 ± 2.85a	32.12 ± 4.23bc
CT	0 ~ 10	1.06 ± 0.01b	1.56 ± 0.32ab	1.56 ± 0.06b	59.34 ± 0.11a	42.98 ± 1.21b	37.82 ± 3.11ab
	11 ~ 20	1.09 ± 0.02ab	1.50 ± 0.08b	1.64 ± 0.05b	58.83 ± 0.54b	43.53 ± 2.87b	39.68 ± 4.29a
	21 ~ 30	1.17 ± 0.05a	1.74 ± 0.04a	1.81 ± 0.12a	55.28 ± 2.00b	34.22 ± 1.61c	31.47 ± 3.16c

CT is conventional tillage; FL is Fenlong tillage; Different lowercase letters indicate significant differences among treatments at *p* < 0.05. Data are presented as the mean ± standard error (SE).

**Table 3:** ANOVA and paired *t*-test of bulk soil chemical properties of cassava under different treatments.

Year	Treatment		pH	OM (g·kg^−1^)	AN (mg·kg^−1^)	AP (mg·kg^−1^)	AK (mg·kg^−1^)
2019	Tillage (T)	*MS*	0.123^ns^	74.15^**^	187.5^**^	4973^***^	174990^***^
	Fertilizer (F)	*MS*	0.068^ns^	6.72^ns^	72.19^**^	553.2^***^	11475^**^
	T × F	*MS*	0.38^ns^	5.06^ns^	64.60^*^	182.5^**^	956.4^ns^
	Paired *t*-text (CT vs. FL)	T value	−1.035	−3.720	−3.075	−9.053	−13.46
		*df*	71	71	71	71	71
		*p*	ns	***	**	***	***
2020	T	*MS*	0.360^ns^	3.312^ns^	454.8^ns^	0.001^ns^	229442^**^
	F	*MS*	0.077^ns^	1.604^ns^	268.0^ns^	592.8^*^	31256^ns^
	T × F	*MS*	0.067^ns^	1.402^ns^	253.2^ns^	161.5^ns^	6347^ns^
	Paired *t*-text (CT vs. FL)	T value	2.323	−1.041	−1.206	0.002	−4.957
		*df*	71	71	71	71	71
		p	*	ns	ns	ns	***

CT is conventional tillage; FL is Fenlong tillage; df denotes freedom; MS represents the mean square, * is significant at the 0.05 level. ** is significant at the 0.01 level. *** is significant at the 0.001 level. ns is not significant.

### 3.2 Effects of tillage and N application rate on bulk soil microbial composition

#### 3.2.1 Alpha diversity of bulk soil microbial communities

A total of 1,292,542 and 1,540,957 high-quality sequences were obtained for the cassava bulk soil bacteria in 2019 and 2020, while 996,844 and 1,075,112 high-quality sequences were obtained for fungi in 2019 and 2020, respectively. The average lengths of the bacterial and fungal sequences were 416 and 381, respectively, with a ≥95% OTU library coverage rate for each treatment. For the bacteria, ANOVA results indicated that variation of the Ace index among treatments was significantly influenced by fertilizer in 2019, while the Simpson index in 2020 was influenced by the interaction of tillage and fertilizer (Supplementary Table S1). The Shannon index of the CT block in 2019 was found to decrease significantly with the N inputs, while the Simpson index showed opposite trends (Supplementary Table S2). According to the Ace and Chao indices, the bacterial communities of the low N input plots (0N and 25N) showed higher richness than those with higher N input treatments (50N and 100N) in 2019. For the soil fungi, neither tillage nor N application affected its alpha diversity (Supplementary Tables S3, S4).

#### 3.2.2 Beta diversity of bulk soil microbial communities

Principal coordinate analysis (PCoA) scatter plots were constructed for soil bacterial (Supplementary Figure S4A,B) and fungal (Supplementary Figures S4C,D) communities based on the Bray-Curtis distance matrix. The results showed that the cumulative explanation rates of PC1 and PC2 was 34.76% (Supplementary Figure S4A) in 2019, while the cumulative explanation rate was 35.28% in 2020 (Supplementary Figure S4B). The Analysis of Similarities (ANOSIM) revealed a significant treatment effect on bacterial community structure in both 2019 and 2020. This finding was visually supported by the scatter plots, in which samples from different treatments were distinctly separated, whereas those from the same treatment clustered together, indicating high within-group consistency in bacterial community composition. For soil fungi, the first-two principal coordinates explained 41.16% (2019) and 53.82% (2020) of the total variance. However, ANOSIM analysis showed no significant differences among treatment groups in either year, with low *R* values (0.02844 in 2019, *p* = 0.32; 0.06581 in 2020, *p* = 0.197). This is consistent with the overlapping distribution of samples observed in the ordination plot.

#### 3.2.3 Community structure and composition of soil microbe

**Figure 2:**
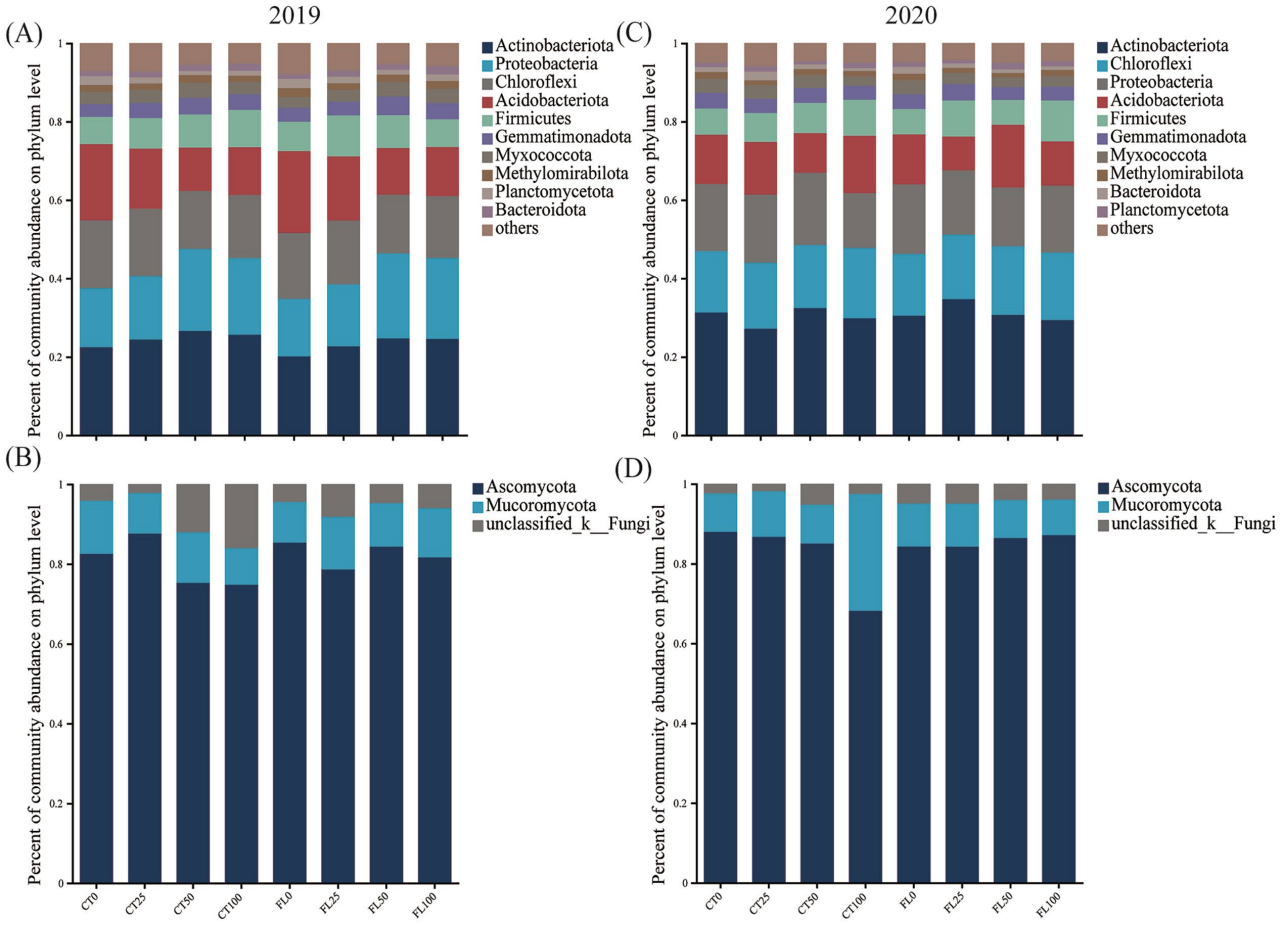
Relative abundances (%) of soil microbial communities at the phylum level (**A,C**: bacteria; **B,D**: fungi).

**Figure 3:**
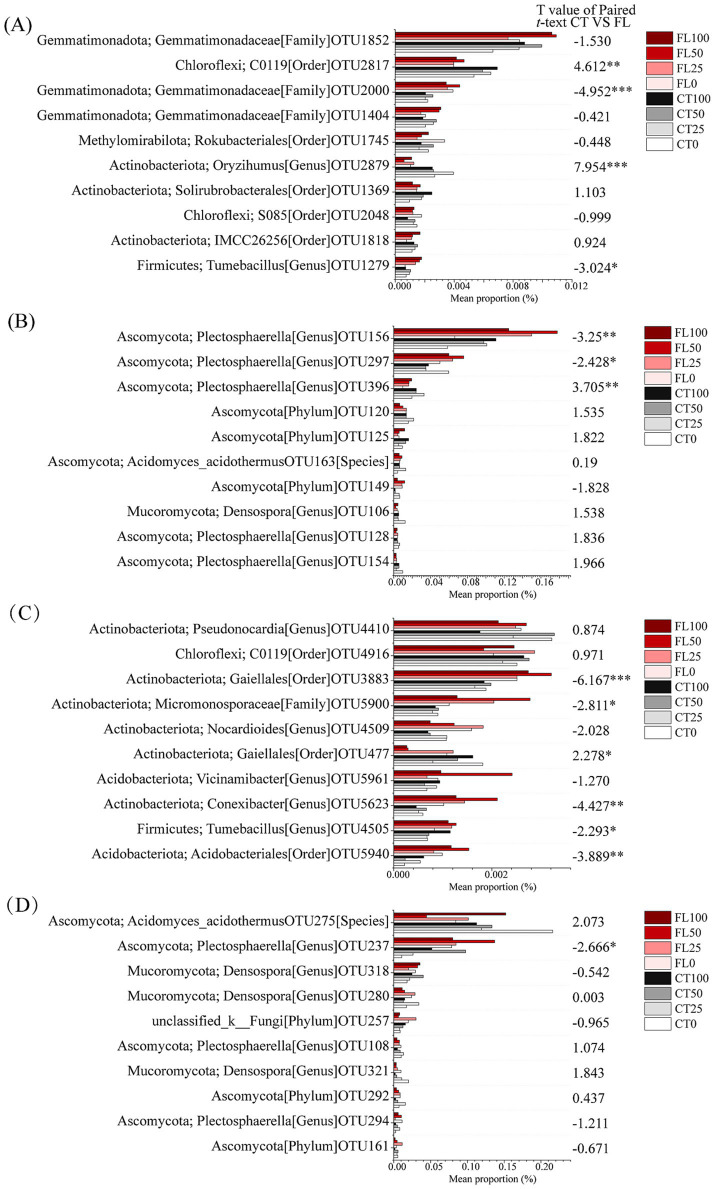
The top 10 significant different OTUs of soil bacteria (**A**, 2019; **C**, 2020) and fungi (**B**, 2019; **D**, 2020) based on comparison of their relative abundances under different treatments and pair *t*-test of their relative abundances between Fenlong tillage (FL) and conventional tillage (CT). * is significant at the 0.05 level. ** is significant at the 0.01 level. *** is significant at the 0.001 level.

Both the bacterial and fungal community compositions at the phylum level of all treatments were shown in Figure 2. The dominant bacterial communities mainly consisted of Actinobacteriota (20.06% ~ 34.67%), Proteobacteria (21.68% ~ 14.70%), Chloroflexi (18.44% ~ 14.00%), Acidobacteriota (20.89% ~ 8.60%), and Firmicutes (10.47% ~ 6.32%). In 2019, the relative abundance of Actinobacteriota and Proteobacteria increased with the nitrogen application rate, whereas the proportions of Chloroflexi and Acidobacteriota decreased (Figure 2A). The top 10 significantly different OTUs of soil bacteria among treatments were listed in Figures 3A,C. Paired *t*-test indicated that the relative abundance of OTU2000 and OTU1279 in 2019 and OTU3883, OTU5900, OTU5623, OTU4505, and OTU5940 in 2020 of the FL group were found higher than that of CT, while the relative abundance of OTU2817 and OTU2879 in 2019 and OTU477 in 2020 of the FL group were lower than that of CT.

The fungal communities of cassava bulk soil mainly consisted of Ascomycota (68.12% ~ 87.97%) and Mucoromycota (14.70% ~ 21.68%). In the CT group, the relative abundance of Ascomycota of lower N inputs (0N and 25N) treatments was higher than that of the high N application treatments (50N and 100N) (Figures 2B,D). However, there was no difference of the relative abundance of Ascomycota among the fertilizer treatments within the FL group. The top 10 significant different OUTs of soil fungi among different treatments were listed in Figures 3B,D. Paired *t*-test revealed that the relative abundance of OUT156 and OUT297 in 2019 and OTU237 in 2020 of the FL group were higher than that of CT, while OTU396 were enriched in CT in 2019 than that of the FL group (Figures 3B,D).

#### 3.2.4 Association analysis of soil microbial community and environmental factors

**Figure 4:**
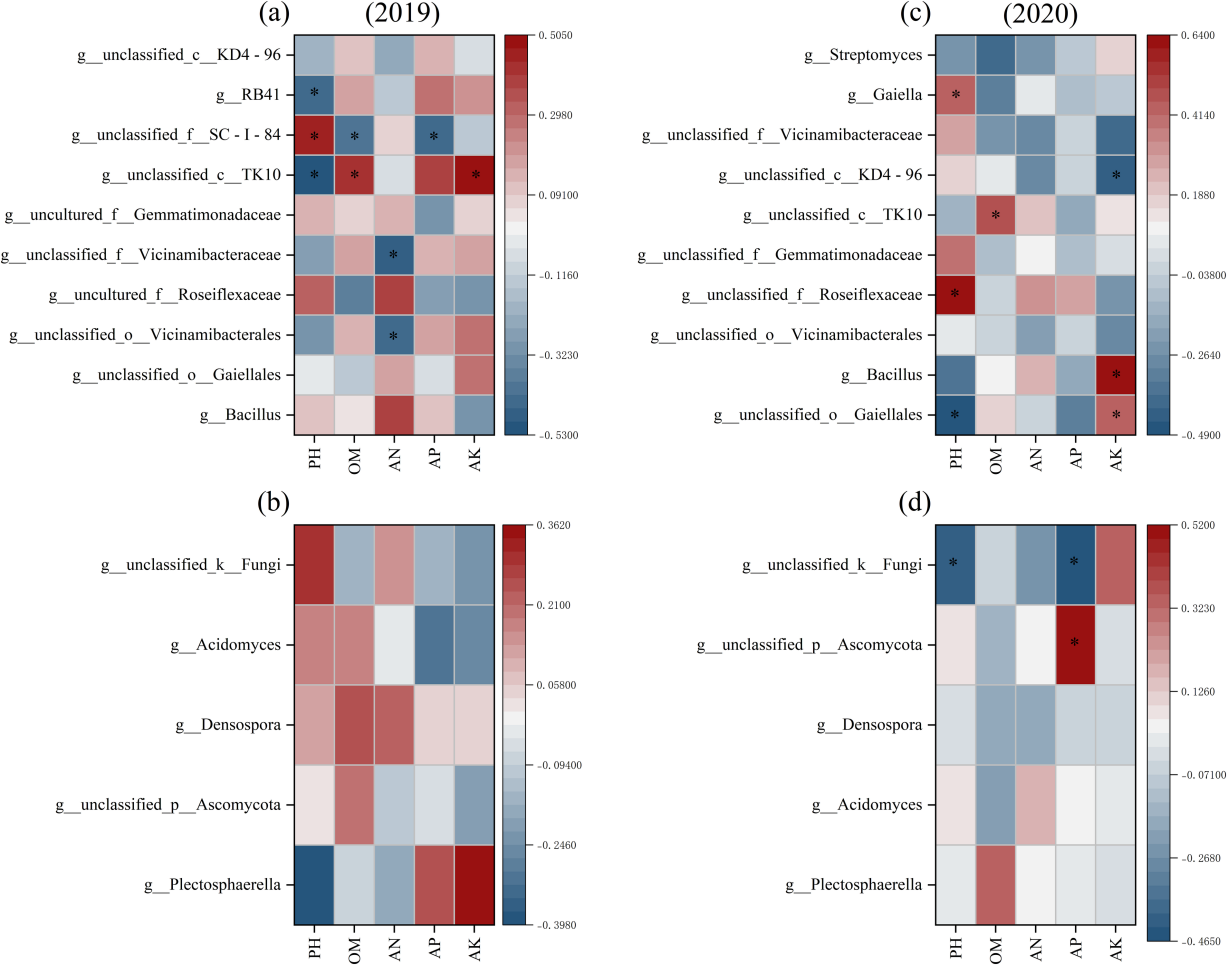
Heatmaps of correlation between soil bacterial (**a** and **b**) and fungal (**c** and **d**) communities at the genus level and soil chemical properties. * Is significant at the 0.05 level, ** is significant at the 0.01 level. The values on the axis indicate R-value.

Spearman correlation heatmaps were plotted based on the top 10 bacterial communities in relative abundance at the genus level and soil chemical factors (Figures 4A,C). Soil pH was positively correlated with *g__unclassified_f__SC-I-84*, *g__unclassified_f__Roseiflexaceae*, and *g__Gaiella*, but negatively correlated with *g__unclassified_c__TK10*, *g__RB41*, and *g__unclassified_o__Gaiellales*. Soil OM exhibited a positive correlation with *g__unclassified_c__TK10* and a negative correlation with *g__unclassified_f__SC-I-84*. Soil AN was significantly negatively correlated with *g__unclassified_o__Vicinamibacterales* and *g__unclassified_f__Vicinamibacteraceae*. Similarly, soil AP showed a significant negative correlation with *g__unclassified_f__SC-I-84*. In contrast, soil AK was positively correlated with *g__unclassified_c__TK10*, *g__unclassified_o__Gaiellales*, and *g__Bacillus*, while negatively correlated with *g__unclassified_c__KD4-96*.

There was no significant correlations found between the dominant genus of the fungal community and soil chemical properties in 2019 (Figure 4B). However, *g__unclassified_k__Fungi* exhibited a significant negative correlation with soil pH and AP, whereas *g__unclassified_p__Ascomycota* was significantly positively correlated with AP.

### 3.3 Effect of tillage and fertilization regime on cassava agronomic traits

ANOVA and paired *t*-tests of cassava agronomic traits revealed that FL had a significant promoting effect on cassava plant height and stem diameter in 2019, and this effect persisted until 2020 (Supplementary Figures S5A,B). In the CT group, higher plant height and larger stem diameter were found in the 75N, 100N, 33NPK, and 66NPK treatments than other treatments in 2019, indicating that N did promote cassava growth, especially when equilibrium regimens of N, P, and K applied (Supplementary Figures S5A,B). Among all fertilized treatments in the FL group, the growth-promoting effects of FL on cassava agronomic traits in 2019 was stronger than fertilizer and thus fertilizer showed little effect on cassava. In 2020, equilibrium fertilization regimens 66NPK and 100N in CT group showed stronger positive effects on cassava plant height and stem diameter than other fertilization rates (Supplementary Figures S5C,D). In the meanwhile, plant height and stem diameter of CK and 25N treatments of the FL group were significantly lower than other higher N input treatments, indicating that N from the first year accumulated in 2020 and showed stronger effects on cassava than the first year (Supplementary Figure S5).

**Figure 5:**
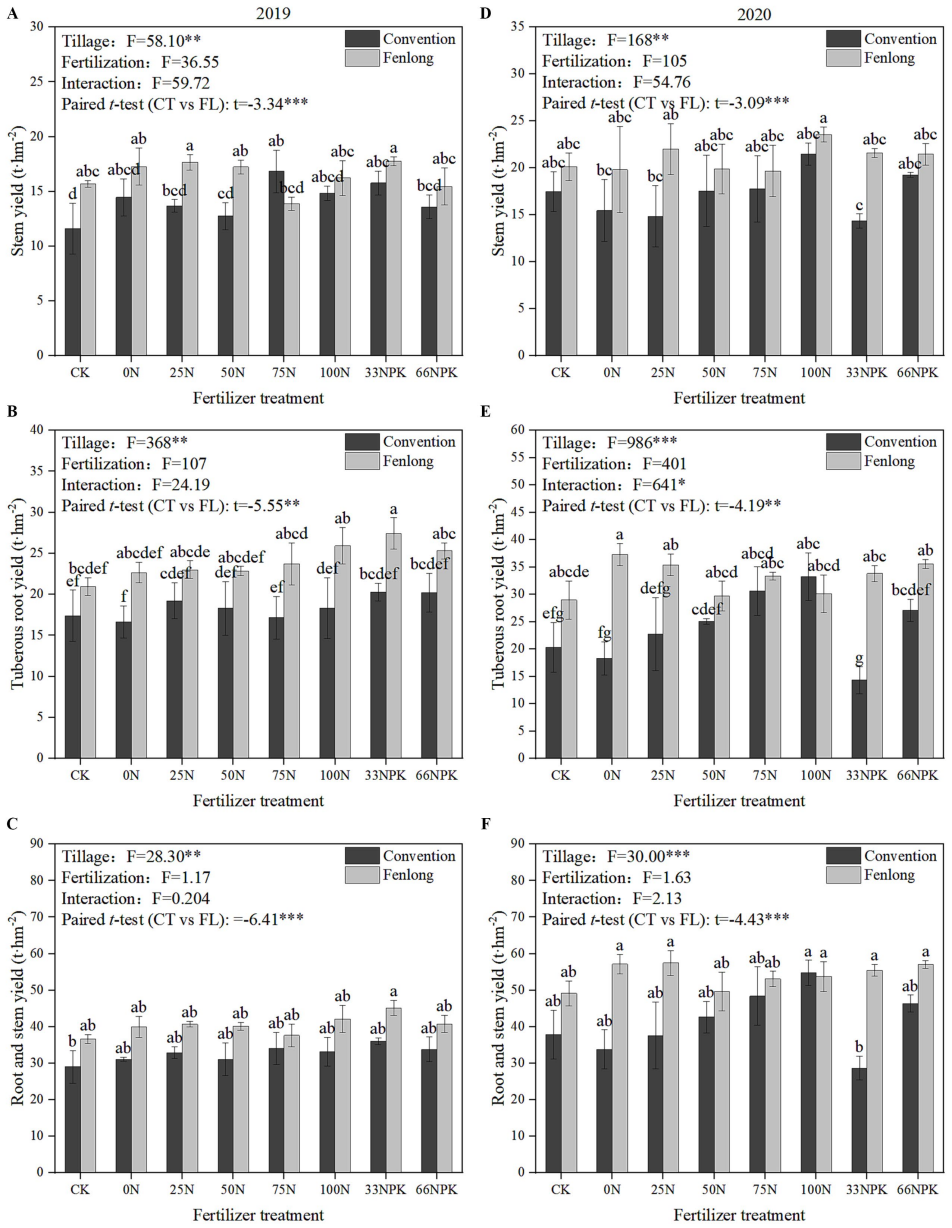
Stem and root yields of cassava under different tillage methods and fertilizer treatments (**A–C** were samples from 2019; **D–F** were samples from 2020). Data are presented as the mean ± standard error (SE).

Tillage treatment also dominantly influenced cassava yields in 2019 and 2020 (Figure 5). In 2019, the stem yields of all the fertilized treatments within the CT group were all higher than CK, while the root and total yields of all fertilized treatments within the FL group were higher than CK. In 2020, the root yield of cassava was significantly influenced by the interaction of tillage treatment and fertilizer regime. The root and yields were increased with the inputs of N within the CT group. During the whole study period, cassava yields under FL were significantly higher than under CT by 9.04 to 135.81%, according to the paired *t*-test results. However, there were no significant difference of yields among all fertilization rates within the FL treatment, suggesting that it was possible to maintain the cassava yield with low fertilizer input when the FL tillage was applied.

### 3.4 Effect of tillage and fertilization regime on the FUE of cassava

**Figure 6:**
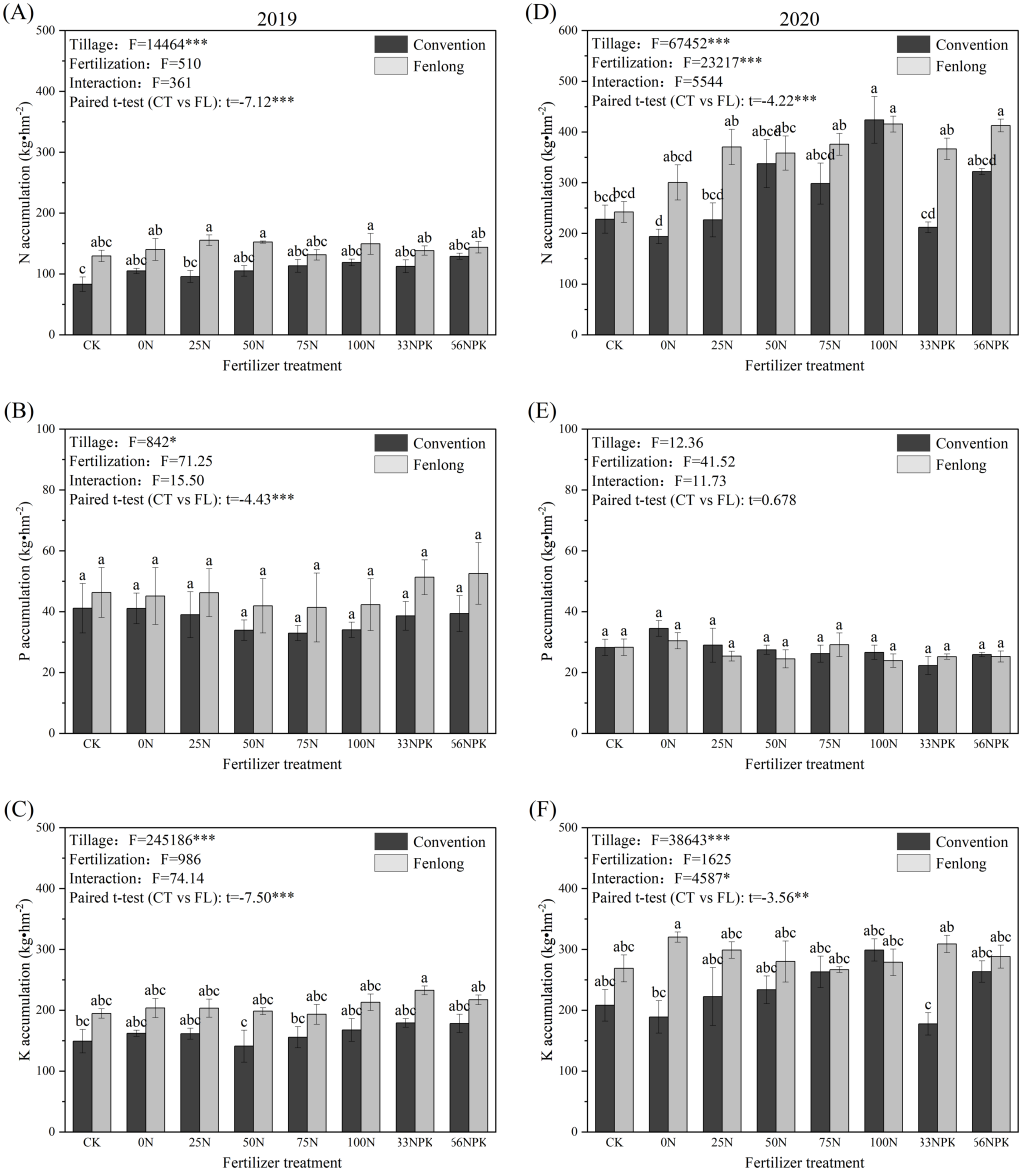
Nutrient element accumulation under different tillage methods and fertilizer treatments (**A–C** were samples from 2019; **D–F** were samples from 2020). Data are presented as the mean ± standard error (SE).

Tillage dominantly influenced the nutrients accumulations of cassava in 2019 and 2020, while fertilization showed significant effects on the N accumulation in 2020 (Figure 6). Compared to the CT group, FL significantly increased the accumulation of N, P, and K in cassava by 11.51% ~ 62.07, 9.86% ~ 33.51, and 21.75% ~ 40.76%, respectively, in 2019 (Figures 6A–C), while the nitrogen accumulation was found increased with N application rates among all fertilized treatments within the CT or FL groups in 2020 (Figure 6D). The interaction between tillage methods and fertilization treatments significantly affected the K accumulation in cassava in 2020, which increased with N application rates within the CT group (Figure 6F). Variations of N and K accumulations in 2020 indicated that FL showed positive effects on cassava yield, leading to a higher nutrient accumulation with lower N inputs than that of CT (Figures 5F, 6D,F).

**Figure 7:**
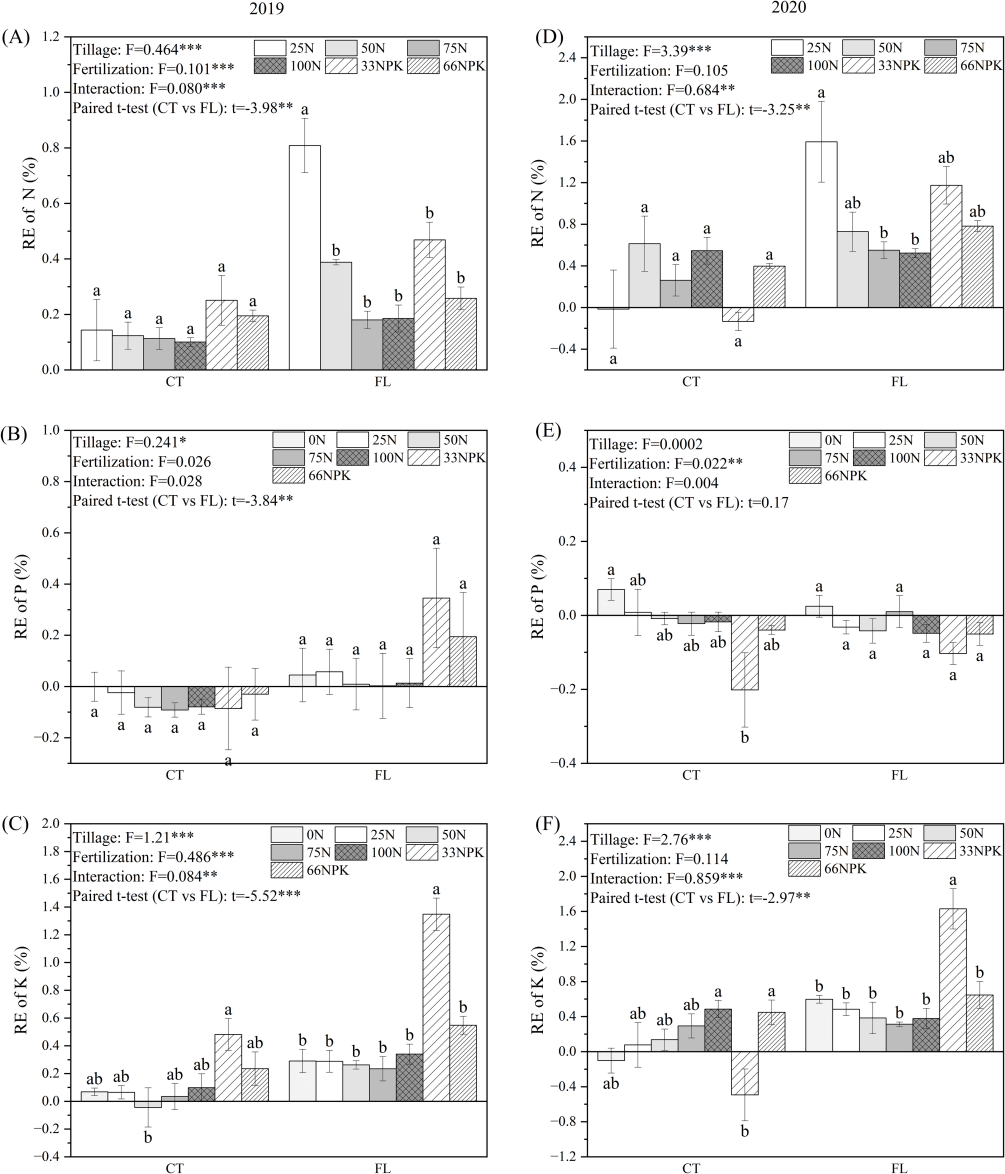
The FUE of N, P, and K of cassava under different tillage methods and fertilization treatments (**A–C** were samples from 2019; **D–F** were samples from 2020). Data are presented as the mean ± standard error (SE).

According to ANOVA results, FUE of N, P, and K of cassava under different treatments were dominantly influenced by tillage treatment, while fertilizer treatment showed significant effects on FUE of N and K in 2019 and FUE of P in 2020 (Figure 7). Paired *t*-test indicated that FUE of N (2019, *t* = −3.98, *p* < 0.01; 2020, *t* = −3.25, *p* < 0.01) and K (2019, *t* = −5.52, *p* < 0.001; 2020, *t* = −2.97, *p* < 0.01) of cassava of the FL group were generally higher than that of CT group within the whole study period. The FUE of N and P decreased with the N inputs. However, when the application amounts of nitrogen, phosphorus, and potassium fertilizers were balanced (33NPK and 66NPK treatment areas), the FUE of nitrogen increased by 19.49 to 117.32%, indicating that a reasonable fertilizer ratio can enhance the nitrogen utilization rate by cassava. Furthermore, in FL group in 2019, FUE of P in the balanced fertilizer treatment plots (33NPK and 66NPK) were 19.42 and 34.53%, respectively, which were higher than other fertilizer treatments (Figures 7B,C).

**Figure 8:**
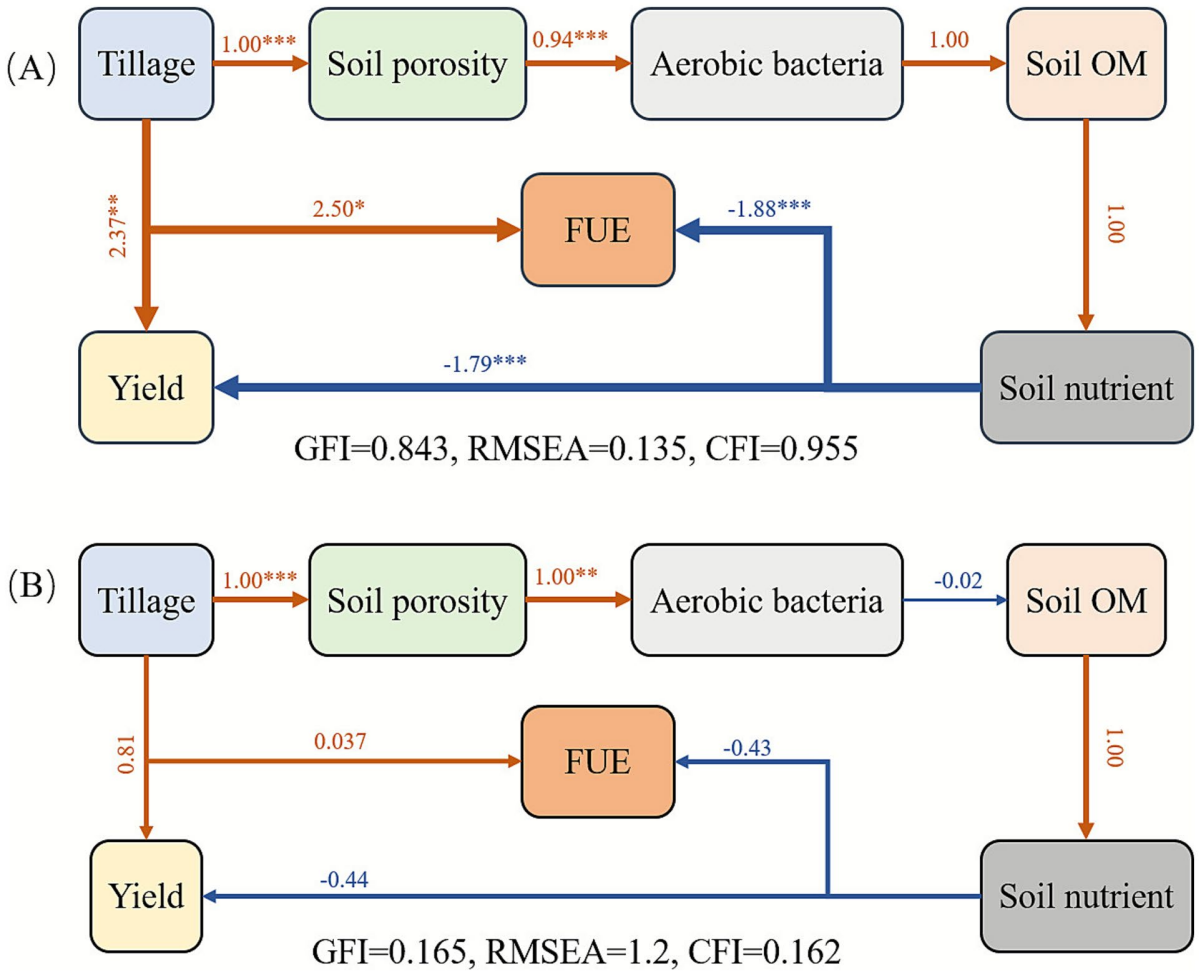
The structural equation model explains the mechanisms by which tillage affect cassava yield and FUE (**A**, 2019; **B**, 2020). The GFI, RMSEA, and CFI in the figure represent the Goodness-of-Fit Index, Root Mean Square Error of Approximation, and Comparative Fit Index, respectively, which are used to assess the model’s goodness of fit. Standardized path coefficients are given next to each arrow. * is significant at the 0.05 level. ** is significant at the 0.01 level. *** is significant at the 0.001 level.

The results of structural equation modeling indicated a clear mechanistic pathway for the year 2019 (Figure 8A): FL improved soil porosity (*β* = 1.00, *p* < 0.001), which subsequently increased the relative abundance of aerobic bacteria (*β* = 0.94, *p* < 0.001), thus facilitating the mineralization of soil organic matter. These processes ultimately resulted in a significant increase in cassava yield (*β* = 2.37, *p* < 0.01) and FUE (*β* = 2.50, *p* < 0.05). Compared with the 2019 model, the 2020 structural equation model showed that the pathways from FL to yield and FUE were no longer significant (Figure 8B). This mechanistic shift can be attributed to the concurrent decline in the effect of FL and the increase in the effect of fertilizer application. Soil physical properties under FL in 2020 further explained this change: bulk density increased (2019, 1.37 ~ 1.53 g·cm^−3^; 2020, 1.55 ~ 1.67 g·cm^−3^) while porosity decreased (2019, 45.25% ~ 47.35%; 2020, 32.12% ~ 43.94%), narrowing the difference in soil aeration between FL and CT (Table 2). Moreover, ANOVA and paired *t*-test results on soil chemical properties in 2020 indicated that tillage methods had no significant influence on soil OM (2019, mean square, *MS* = 74.15**, *t* = −3.720***; 2020, *MS* = 3.312^ns^, *t* = −1.041^ns^), AN (2019, *MS* = 187.5**, *t* = −3.075**; 2020, *MS* = 454.8^ns^, *t* = −1.206^ns^), AP (2019, *MS* = 4973***, *t* = −9.053***; 2020, *MS* = 0.001^ns^, *t* = 0.002^ns^), P accumulation (2019, *MS* = 842*, *t* = −4.43***; 2020, *MS* = 12.36^ns^, *t* = 0.678^ns^), or FUE-P (2019, *MS* = 0.241*, *t* = −3.84**; 2020, *MS* = 0.0002^ns^, *t* = 0.17^ns^) (Table 3, Figures 6, 7). Together, the diminished influence of FL on soil properties and the enhanced effect of fertilizer application in 2020 ultimately led to the non-significance of the pathways from FL to yield and FUE. Furthermore, a PCA model was developed to identify the optimal fertilization regime under FL tillage. Composite scores were calculated for all fertilizer treatments, with the 33NPK treatment achieving the highest score (Table 4), thereby establishing it as the optimal fertilization regime under the FL system.

**Table 4:** Composite score for all fertilizer treatments under FL tillage.

Treatment	Composite score	Rank
CK	7.8147	8
0N	8.8258	5
25N	9.5441	2
50N	8.7739	6
75N	8.4256	7
100N	9.1938	4
33NPK	10.4235	1
66NPK	9.3002	3

## 4 Discussion

### 4.1 Effects of tillage and fertilizer regime on soil physicochemical properties

Tillage can directly change the physical properties of soil, thus affecting chemical properties and microbial communities, creating favorable environments for crop growth (Ahmadi et al., 2024; Adeleke et al., 2023; Chichongue et al., 2020). Applying optimum tillage techniques combined with fertilizer application does not only improve soil quality but also results in higher crop production (Vilakazi et al., 2022). In the current study, the FL treatment showed lower soil bulk density and higher porosity than the CT plots, especially at the 21 ~ 30 cm layer (Table 2). This finding was consistent with previous researches (Zhai et al., 2017, 2019). Our previous study showed that the clay and the silt of cassava rhizosphere soil were also influenced by FL, fertilization, and their interaction (Li et al., 2023). For bulk soil chemical properties, tillage showed stronger effects on nutrient parameters than the fertilizer treatment, and the contents of OM, AN, AP, and AK of soil samples under the FL treatment were significantly higher than that of the CT treatment (Table 3). The same phenomenon was also found in cassava rhizosphere soils under FL and CT treatments, and tillage treatment also showed stronger influence on the chemical properties of cassava rhizosphere soils than N applications (Li et al., 2023). According to the climate data (Figure 1), waterlogging occurred during the summer time, especially in June of 2020. A lower bulk density and higher porosity of the bulk soil under FL treatment enhanced the infiltration of the rainfall, thus increasing the resistance to waterlogging stress of cassava. The aerobic conditions in soil created by FL can also accelerate the oxidation of reduced N compounds to nitrate, which is susceptible to leaching out of the soil due to its high solubility. Loss of nitrate as coupled ions promoted cation (Ca^2+^ and Mg^2+^) leaching, which can accelerate the soil acidification in FL treatment, ending up with a lower pH value than CT in 2020 (Table 3, Supplementary Figure S3) (Zhou et al., 2024). However, the high solubility of nitrate in FL treatment would be also benefit the N absorbency for cassava under drought stress, leading to a higher N accumulation in cassava than that of CT (Figure 6).

### 4.2 Effects of tillage and N application rates on soil microbial composition

Soil microbiomes encompass a great diversity of organisms that perform vital ecological functions, including nitrogen and carbon cycling (García-Serquén et al., 2024), and their diversity positively correlates with multiple ecosystem functions such as plant productivity, nutrient cycling, and decomposition (Wu et al., 2024). In the current study, soil physical and chemical factors varied with both tillage and the fertilization regimes (Tables 2, 3), leading to a shifting of the soil microbe structure (Figure 2), which was consistent with previous reports (Chi et al., 2024; Özbolat et al., 2023; Ouverson et al., 2021). Since the field had been abandoned for 2 years before the experiment, the initial soil microbial species were rich and diverse. The application of nitrogen fertilizer reduced the Shannon, Ace, and Chao indices while increasing the Simpson index of soil microbes (Supplementary Table S2). This suggests that nitrogen input altered soil properties such as pH, OM, and AN content, ultimately leading to decreased richness and diversity in both bacterial and fungal communities (Table 3, Supplementary Figure S3).

Bacteria and fungi are key decomposers in the soil ecosystem, directly driving the carbon cycle through the breakdown of organic residues (Kramer and Gleixner, 2008; Strickland et al., 2009). Their necromass subsequently constitutes a primary source of stable soil organic matter (Bardgett and van der Putten, 2014). The dominant phyla of the bulk soil bacteria among all treatments in 2019 and 2020 were Actinobacteria, Proteobacteria, Chloroflexi, Acidobacteria, and Firmicutes (Figures 2A,C), which was similar to previous results (Jia et al., 2024; Nimnoi et al., 2024). Actinobacteria and Proteobacteria are considered as key roles in the decomposition of organic matter in soil, while organic C provides energy resource for microbial growth in soil (Zhao et al., 2019; Xiao et al., 2016). Under both FL and CT treatments in 2019, the relative abundance of Actinobacteria and Proteobacteria increased with the amount of nitrogen applied. In 2020, however, the relative abundance of Actinobacteria in all fertilized treatments was consistently higher than that in 2019 (Figure 2). This pattern aligns with previous findings indicating that the richness of Actinobacteria is negatively correlated with soil pH and can serve as an indicator of soil acidification induced by long-term nitrogen fertilization (Ren et al., 2020). Proteobacteria, a key component of soil bacteria that includes nitrogen-fixing and pathogenic species, showed a decrease in relative abundance in 2020. This decline may be attributed to the concurrent increase in Actinobacteria, which likely outcompeted Proteobacteria for carbon resources to support their own population growth.

Furthermore, FL treatment consistently maintained higher soil porosity than CT (Table 2), creating a more favorable aeration environment for aerobic bacteria. Correspondingly, it promoted the enrichment of several predominantly aerobic taxa, including OTU2000 (Gemmatimonadaceae, Family) and OTU1279 (*Tumebacillus, Genus*) in 2019, and OTU5900 (Micromonosporaceae, Family), OTU4505 (*Tumebacillus, Genus*), OTU5623 (*Conexibacter, Genus*), and OTU5940 (Acidobacteriales, Family) in 2020 (Figures 3A,C). Previous studies have confirmed the aerobic nature of these taxa (Ivanitskaia et al., 1982; Lei et al., 2023; Mujakic et al., 2023; Zhang et al., 2023). Aerobic bacteria contribute significantly to soil carbon cycling (Angélica et al., 2024), which likely accelerated nutrient release and led to higher contents of AN, AP, and AK under FL treatment compared to CT (Table 3). This suggests that FL tillage enhances the soil C/N cycling pathway—a key mechanism for maintaining soil ecosystem stability (Gao et al., 2025). Notably, OTU5623, identified as a strictly aerobic *Conexibacter*, can reduce nitrate to nitrite and may play an important role in nitrification (Pukall et al., 2010; Monciardini et al., 2003). Its enrichment under FL treatment could further support the regulation of soil nitrogen cycling.

Fungi regulate the balance of carbon and nutrients in the soil ecosystem by metabolizing and decomposing complex organic matter (Žifčáková et al., 2016). Ascomycota and Mucoromycota were dominant phyla in the bulk soil fungal communities among all treatments in current study (Figures 2B,D), which can decompose refractory organic matter in soil and play important roles in nutrient cycling (Zhao et al., 2023; Das et al., 2019). Ascomycota growth is primarily regulated by soil nitrogen content, with high N levels generally suppressing its abundance (Zhang et al., 2024; Fontaine et al., 2011). Consistent with this, our study observed that high N inputs significantly inhibited Ascomycota growth in the CT group throughout the experimental period (Figures 2B,D). However, the relative abundance of Ascomycota remained relatively stable across fertilized treatments within the FL group. This stability may be attributed to the ability of FL to improve bulk soil physicochemical properties, particularly by maintaining higher AN content, thereby helping to preserve the structure of the fungal community. Furthermore, FL tillage simultaneously created more stable soil conditions for Mucoromycota. These fungi play a key role in soil carbon cycling due to their strong capacity for decomposing OM—a function essential for maintaining soil fertility and microbial diversity. As important regulators of nutrient and carbon cycles, Mucoromycota significantly influence terrestrial ecosystems by shaping soil structure and function (Van der Heijden et al., 2015). In summary, compared to CT, FL treatment promoted greater stability in the soil microbial structure. This enhanced stability improved the ecosystem’s resistance to external disturbances, such as nitrogen stress, supporting FL as a beneficial practice for sustaining soil ecological functions.

### 4.3 Effects of tillage and fertilizer regimes on cassava and its FUE

As we mentioned before, tillage directly changes the soil physical properties, while soil microbes directly and indirectly influence crop productivity and its nutrient acquisition ability by changing the nutrients or stimulating the plant growth (Wang et al., 2021). An increase of nitrogen fertilizer application will accelerate soil acidification, which could enhance the activity of ammonifying bacteria leading to an increase of the volatilization of ammonia nitrogen (Dancer et al., 1973). Additionally, the population of nitrifying bacteria would be influenced under the low pH environment, leading to a decrease of nitrate formation rate, thereby restricting plant’s nitrogen acquisition ability (Saratchandra, 1978). Thus, crop yield and N accumulation increased with the amount of applied nitrogen, while the FUE decreases. FL tillage significantly improved soil conditions for both cassava root development (Figure 5) and microbial activity, particularly that of aerobic microorganisms (Figure 3), by thoroughly loosening the soil and increasing its porosity. This effect was especially pronounced in the 11–30 cm soil layer (Table 2). The resulting soil structure facilitated deeper root penetration compared to CT, enabling more efficient uptake of nutrients and water from subsurface soil layers (Xiao et al., 2023). The well-developed cassava root system under FL treatment was conducive to recruiting rhizosphere microorganisms, which in turn affected soil enzyme activities (Huang et al., 2025; Li et al., 2023). It also reduced the emission of the greenhouse gases, such as CO_2_, N_2_O and CH_4_, and thus improved the fixation abilities of nitrogen and carbon in soil (Xiao et al., 2023; Zhu B. et al., 2023, Zhu S. et al., 2023; Duan et al., 2022).

In addition, the contents of AN, AP, and AK in soil under FL treatment were higher than that of CT (Table 3), offering more nutrients for cassava leading to a higher yield and nutrient accumulation (Figures 5, 6), which was consistent with studies for rice (Zhang et al., 2021), sugarcane (Xiao et al., 2023), and tobacco (*Nicotiana tabacum* L.) (Zhu B. et al., 2023; Zhu S. et al., 2023). The FUE-N and FUE-K of cassava within the FL group were also generally higher than that of CT group (Figure 7), indicating that FL enhances the utilization of nitrogen and potassium fertilizers by cassava.

Furthermore, identifying the optimal fertilization rate under the FL tillage system is critical for sustainable cassava production. Under FL treatment, the highest yield and FUE-K were all found in the 33NPK plot in 2019, while its FUE-N was lower than the 25NPK treatment but slightly higher than others treatments (Figure 7, Table 4). These superior performance of the 33NPK treatment of FL group lasted to 2020, which indicate that FL with 33NPK treatment strikes an ideal balance between cassava yield and FUE among all treatments. Although we did not identify specific functional genes through metagenomics, a significant influence of tillage practices on soil enzyme activities has been demonstrated in our previous work, particularly the urease activity was found notably promoted under FL tillage (Huang et al., 2025). Considering the effects on soil physicochemical properties and microbial communities caused by FL (Figure 8), structural equation models confermed that FL can accelerates the mineralization process of OM by increasing the abundance of aerobic bacteria, thus releases the nutrients from the soil for cassava growth, which ultimately leads to concurrent improvements in both yield and FUE of cassava.

## 5 Conclusion

As a key starch and biofuel feedstock, cassava is predominantly cultivated on marginal land under continuous conventional tillage in southern China, leading to progressive soil compaction and yield decline. This study evaluates the combined effects of FL tillage and fertilization regimes on cassava productivity and soil health, aiming to address the issues of falling FUE and soil acidification linked to excessive N application. Results demonstrated that soil bulk density of the 11 ~ 30 cm layers in 2019 and the 21 ~ 30 cm layer in 2020 under the FL treatment were significantly lower than that of the CT group. Soil porosity of the FL treatment was generally higher than that of the CT group. Soil chemical properties were affected by both tillage and fertilizer treatments. Various aerobic bacteria (OTU2000 and OTU1279 in 2019, OTU5900, OTU4505, OTU5623, and OTU5940 in 2020) were found higher enriched in FL group than that of CT. Tillage dominantly influenced the tuberous root yields and the nutrient accumulation of cassava in 2019 and 2020. The FUE of N and K of cassava within the FL group were significantly higher than that of CT group. Under the FL treatment, the chemical fertilizer application rate as N 118.2 kg·ha^−1^, P_2_O_5_ 29.4 kg·ha^−1^, and K_2_O 61.9 kg·ha^−1^ strikes an ideal balance between cassava yield and FUE.

## Data Availability

The original contributions presented in the study are included in the article/Supplementary material, further inquiries can be directed to the corresponding authors.
