# Supplementary material for: Enhancing cassava N/K use efficiency through Fenlong tillage: driving forces of soil porosity improvement and aerobic bacteria enrichment
Source: Front Microbiol. 2026 Feb 11;17:1739897. doi: 10.3389/fmicb.2026.1739897 (PMC12932608; doi:10.3389/fmicb.2026.1739897)
Supplement: Supplementary file 1 [file Data_Sheet_1.docx]

Supplementary Material

**Table S1** ANOVA and paired *t*-test of alpha diversity index of soil bacteria under different treatment.

**Table S2** Alpha diversity index of soil bacteria under different treatments.

**Table S3** ANOVA and paired *t*-test of alpha index of soil fungi under different treatments.

**Table S4** Alpha diversity index of soil fungi under different treatments.

**Fig. S1** The map of the locations of the sample collection.

**Fig. S2** Layout of different treatments in the field experiment. CT represents conventional tillage, FL represents Fenlong tillage, numbers indicate biological replicates, and capital letters indicate different fertilization treatments.

**Fig. S3** Soil chemical properties under different treatments in 2019 (A, B, C, D, and E) and 2020 (F, G, H, I, and J).

**Fig. S4** Scatter plots of principal coordinates analysis (PCoA) of soil bacterial (A and B) and fungal (C and D) communities based on Bray-Curtis distance.

**Fig. S5** Cassava plant height and stem diameter of 2019 (A and B) and 2020 (C and D) under different treatments.

**Fig. S6** Relative abundances (%) of soil fungal community composition at the class and genus levels in 2019 (A and B) and 2020 (C and D).

**Table S1** ANOVA and paired *t*-test of alpha diversity index of soil bacteria under different treatment

| Treatment |  | 2019 | | | |  | 2020 | | | |
| --- | --- | --- | --- | --- | --- | --- | --- | --- | --- | --- |
|  |  | Diversity indices | | Richness estimators | |  | Diversity indices | | Richness estimators | |
|  |  | Shannon | Simpson | Ace | Chao |  | Shannon | Simpson | Ace | Chao |
| Tillage (T) | MS | 0.00033 | 1.74×10^-7^ | 2.917 | 3521 |  | 0.008 | 5.60×10^-7^ | 15519 | 9572 |
| Fertilizer (F) | MS | 0.017 | 4.13×10^-7^ | 363615* | 145300 |  | 0.014 | 3.13×10^-7^ | 39159 | 36365 |
| T × F | MS | 0.010 | 7.78×10^-7^ | 5551 | 4772 |  | 0.026 | 7.90×10^-7^* | 38561 | 51945 |
| Paired *t*-text  CT VS FL | T value | -0.225 | 0.223 | 0.006 | -0.351 |  | 0.864 | -1.390 | 0.844 | 0.663 |
|  | df | 11 | 11 | 11 | 11 |  | 11 | 11 | 11 | 11 |
|  | p | ns | ns | ns | ns |  | ns | ns | ns | ns |

Note: CT is conventional tillage; FL is Fenlong tillage; *df* denotes freedom; *MS* represents the mean square, * is significant at the 0.05 level. ** is significant at the 0.01 level. *** is significant at the 0.001 level. ns is not significant.

**Table S2** Alpha diversity index of soil bacteria under different treatments

| Year | Tillage | Fertilization | Diversity indices | |  | Richness estimators | |
| --- | --- | --- | --- | --- | --- | --- | --- |
|  |  |  | Shannon | Simpson |  | Ace | Chao |
| 2019 | CT | 0N | 6.82±0.017a | 0.0028±0.00004b |  | 4027±36ab | 4017±46a |
|  |  | 25N | 6.77±0.036ab | 0.0030±0.0002b |  | 4207±378ab | 3954±179a |
|  |  | 50N | 6.68±0.038ab | 0.0034±0.0002ab |  | 3755±121ab | 3733±143a |
|  |  | 100N | 6.62±0.064b | 0.0041±0.0005a |  | 3754±112ab | 3755±151a |
|  | FL | 0N | 6.74±0.028ab | 0.0031±0.00005b |  | 3994±44ab | 4013±16a |
|  |  | 25N | 6.76±0.020ab | 0.0035±0.0003ab |  | 4295±199a | 4062±80a |
|  |  | 50N | 6.68±0.11ab | 0.0035±0.0005ab |  | 3712±196b | 3736±195a |
|  |  | 100N | 6.74±0.046ab | 0.0030±0.00003b |  | 3739±159ab | 3745±98a |
| 2020 | CT | 0N | 6.66±0.05abc | 0.0034±0.0002bc |  | 4245±111ab | 4212±93ab |
|  |  | 25N | 6.79±0.07a | 0.0030±0.0002c |  | 4458±123a | 4430±114a |
|  |  | 50N | 6.62±0.1abc | 0.0036±0.0006abc |  | 4322±127ab | 4284±115ab |
|  |  | 100N | 6.61±0.06bc | 0.0035±0.0002abc |  | 4310±105ab | 4250±94ab |
|  | FL | 0N | 6.75±0.02ab | 0.0031±0.0001c |  | 4416±86a | 4433±104a |
|  |  | 25N | 6.56±0.05c | 0.0043±0.0003a |  | 4346±92ab | 4249±114ab |
|  |  | 50N | 6.64±0.01abc | 0.0035±0.0001abc |  | 4267±60ab | 4251±87ab |
|  |  | 100N | 6.58±0.01bc | 0.0040±0.0001ab |  | 4104±37b | 4084±72b |

Note: CT is conventional tillage; FL is Fenlong tillage; Different lowercase letters indicate significant differences among treatments at *p* < 0.05. Data are presented as the mean ± standard error (SE).

**Table S3** ANOVA and paired *t*-test of alpha index of soil fungi under different treatments

| Treatment |  | 2019 | | | |  | 2020 | | | |
| --- | --- | --- | --- | --- | --- | --- | --- | --- | --- | --- |
|  |  | Diversity indices | | Richness estimators | |  | Diversity indices | | Richness estimators | |
|  |  | Shannon | Simpson | Ace | Chao |  | Shannon | Simpson | Ace | Chao |
| Tillage (T) | MS | 0.045 | 0.001 | 224.3 | 67.02 |  | 0.347 | 0.024 | 2.987 | 11.82 |
| Fertilizer (F) | MS | 0.023 | 0.001 | 1499 | 1347 |  | 0.202 | 0.006 | 378.4 | 762.3 |
| T × F | MS | 0.054 | 0.003 | 994.7 | 1400 |  | 0.057 | 0.004 | 573.0 | 292.6 |
| Paired *t*-text  CT VS FL | T value | -0.685 | 0.407 | -0.604 | -0.292 |  | -1.926 | 2.059 | -0.069 | -0.119 |
|  | df | 11 | 11 | 11 | 11 |  | 11 | 11 | 11 | 11 |
|  | p | ns | ns | ns | ns |  | ns | ns | ns | ns |

Note: CT is conventional tillage; FL is Fenlong tillage; *df* denotes freedom; *MS* represents the mean square, * is significant at the 0.05 level. ** is significant at the 0.01 level. *** is significant at the 0.001 level. ns is not significant.

**Table S4** Alpha diversity index of soil fungi under different treatments

| Year | Tillage | Fertilization | Diversity indices | |  | Richness estimators | |
| --- | --- | --- | --- | --- | --- | --- | --- |
|  |  |  | Shannon | Simpson |  | Ace | Chao |
| 2019 | CT | 0N | 2.88±0.13a | 0.150±0.026a |  | 255±8a | 259±8a |
|  |  | 25N | 2.66±0.21a | 0.178±0.048a |  | 201±20b | 202±22b |
|  |  | 50N | 2.62±0.16a | 0.159±0.021a |  | 215±13ab | 216±12ab |
|  |  | 100N | 2.62±0.2a | 0.168±0.031a |  | 215±18ab | 213±18ab |
|  | FL | 0N | 2.69±0.32a | 0.195±0.07a |  | 238±25ab | 231±26ab |
|  |  | 25N | 2.92±0.13a | 0.119±0.017a |  | 238±18ab | 242±14ab |
|  |  | 50N | 2.78±0.08a | 0.138±0.012a |  | 203±13b | 202±9b |
|  |  | 100N | 2.72±0.13a | 0.162±0.017a |  | 232±15ab | 230±10ab |
| 2020 | CT | 0N | 2.35±0.18ab | 0.239±0.055ab |  | 182±6ab | 179±3a |
|  |  | 25N | 2.55±0.12ab | 0.209±0.044ab |  | 179±16ab | 189±23a |
|  |  | 50N | 2.72±0.13a | 0.156±0.022b |  | 193±6ab | 192±4a |
|  |  | 100N | 2.13±0.36b | 0.292±0.094a |  | 178±19ab | 175±21a |
|  | FL | 0N | 2.77±0.11a | 0.150±0.021b |  | 186±4ab | 185±3a |
|  |  | 25N | 2.79±0.12a | 0.150±0.022b |  | 205±15a | 208±18a |
|  |  | 50N | 2.69±0.1a | 0.161±0.016ab |  | 175±4ab | 180±6a |
|  |  | 100N | 2.47±0.15ab | 0.180±0.023ab |  | 168±8b | 168±12a |

Note: CT is conventional tillage; FL is Fenlong tillage; Different lowercase letters indicate significant differences among treatments at *p* < 0.05. Data are presented as the mean ± standard error (SE).


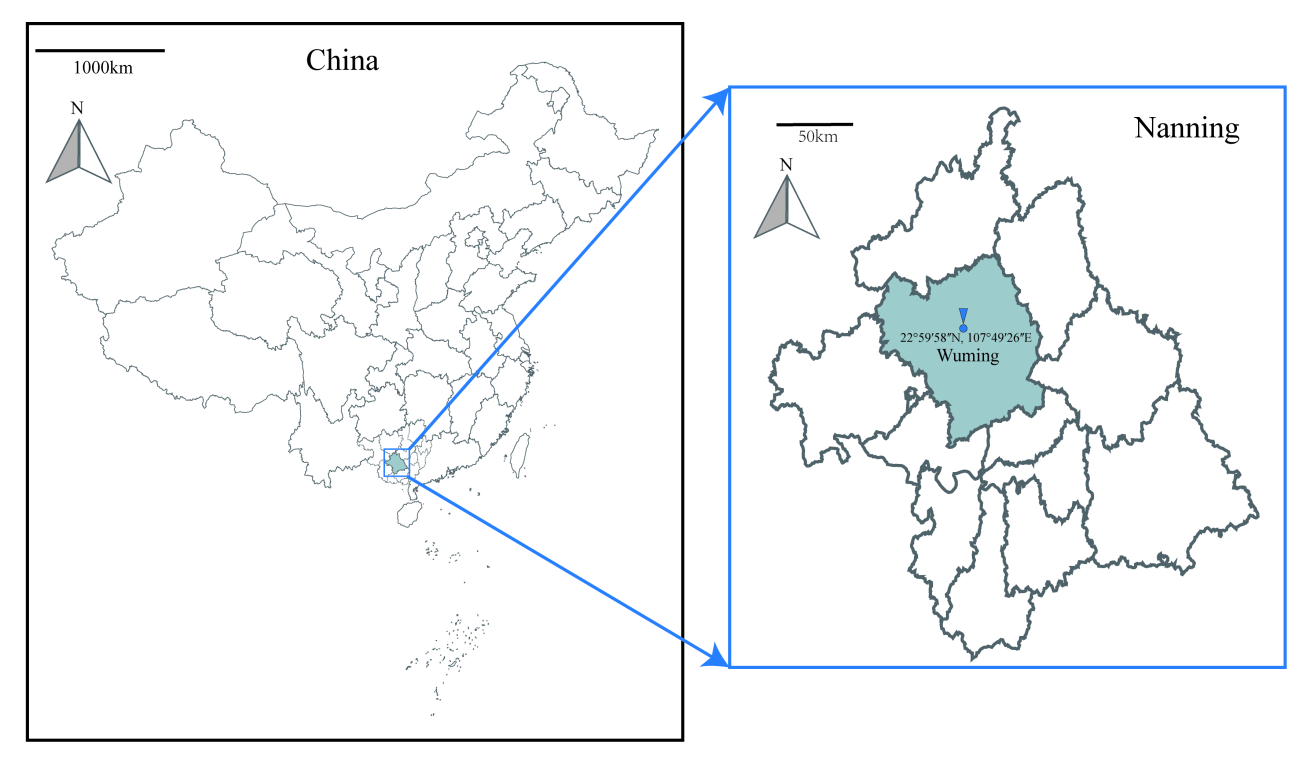


**Fig. S1** Geographic Location of the Experimental Site in China.


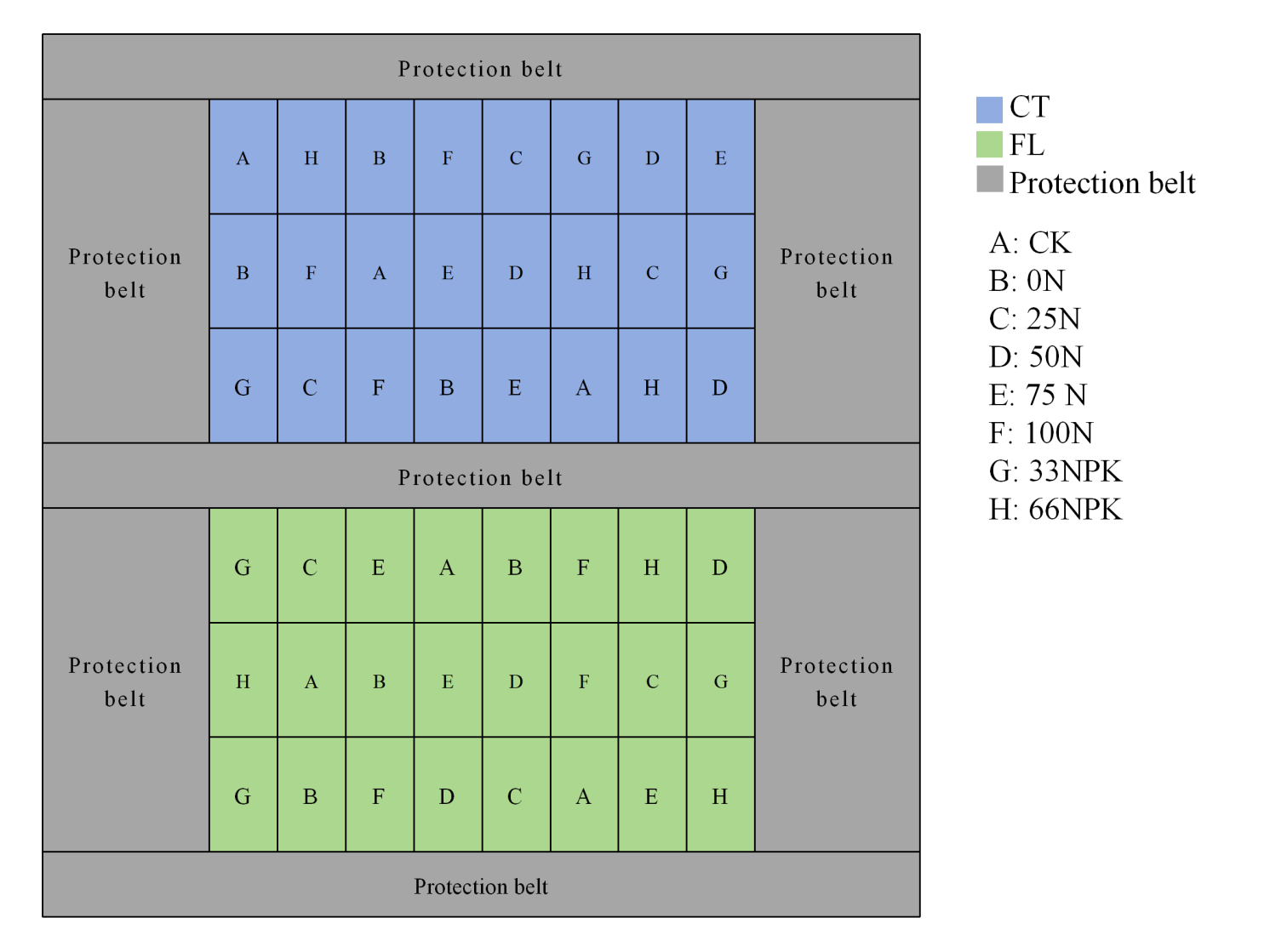


**Fig. S2** Layout of different treatments in the field experiment. CT represents conventional tillage, FL represents Fenlong tillage, numbers indicate biological replicates, and capital letters indicate different fertilization treatments.


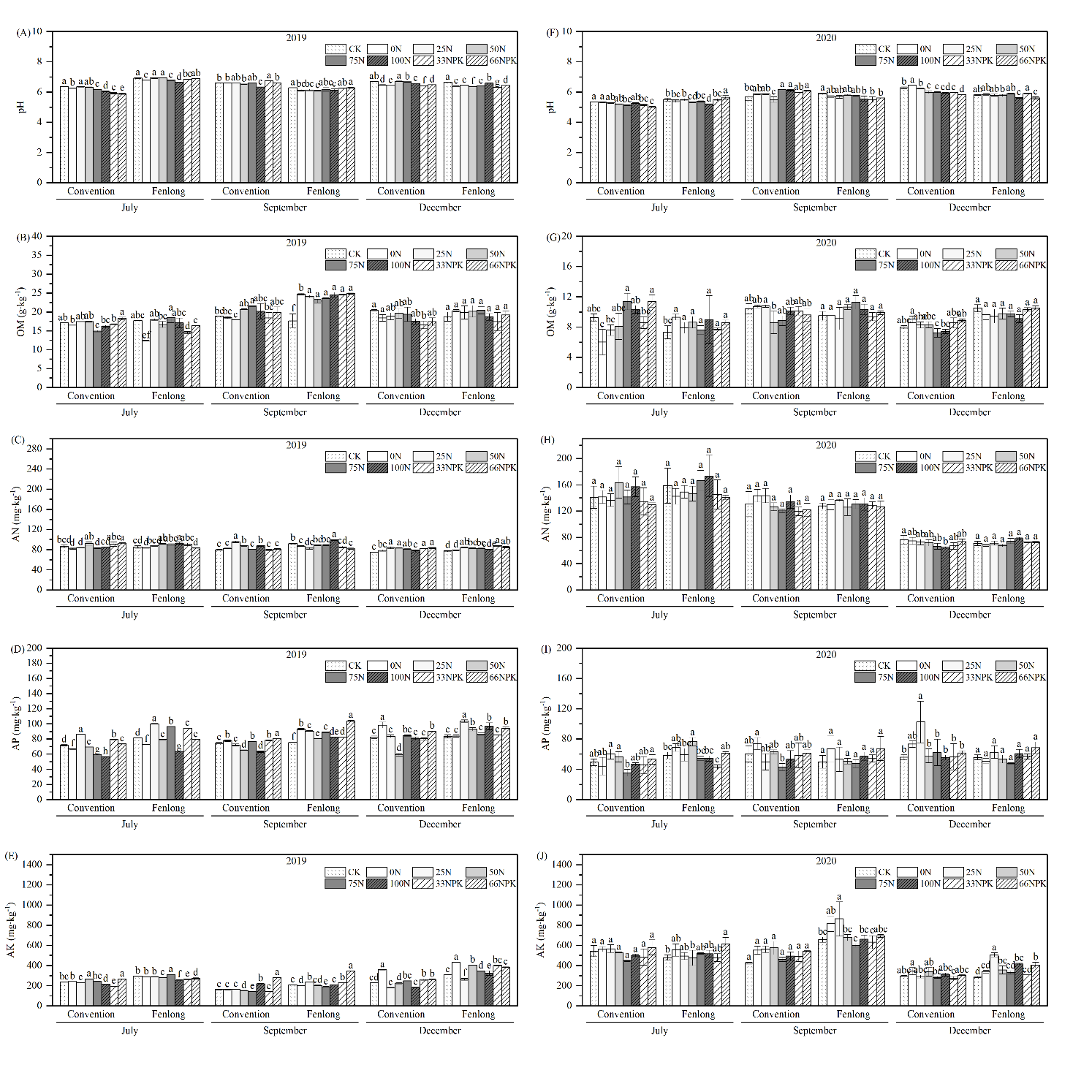


**Fig. S3** Soil chemical properties under different treatments in 2019 (A, B, C, D, and E) and 2020 (F G, H, I, and J). Different lowercase letters indicate significant differences among treatments at *p* < 0.05. Data are presented as the mean ± standard error (SE).


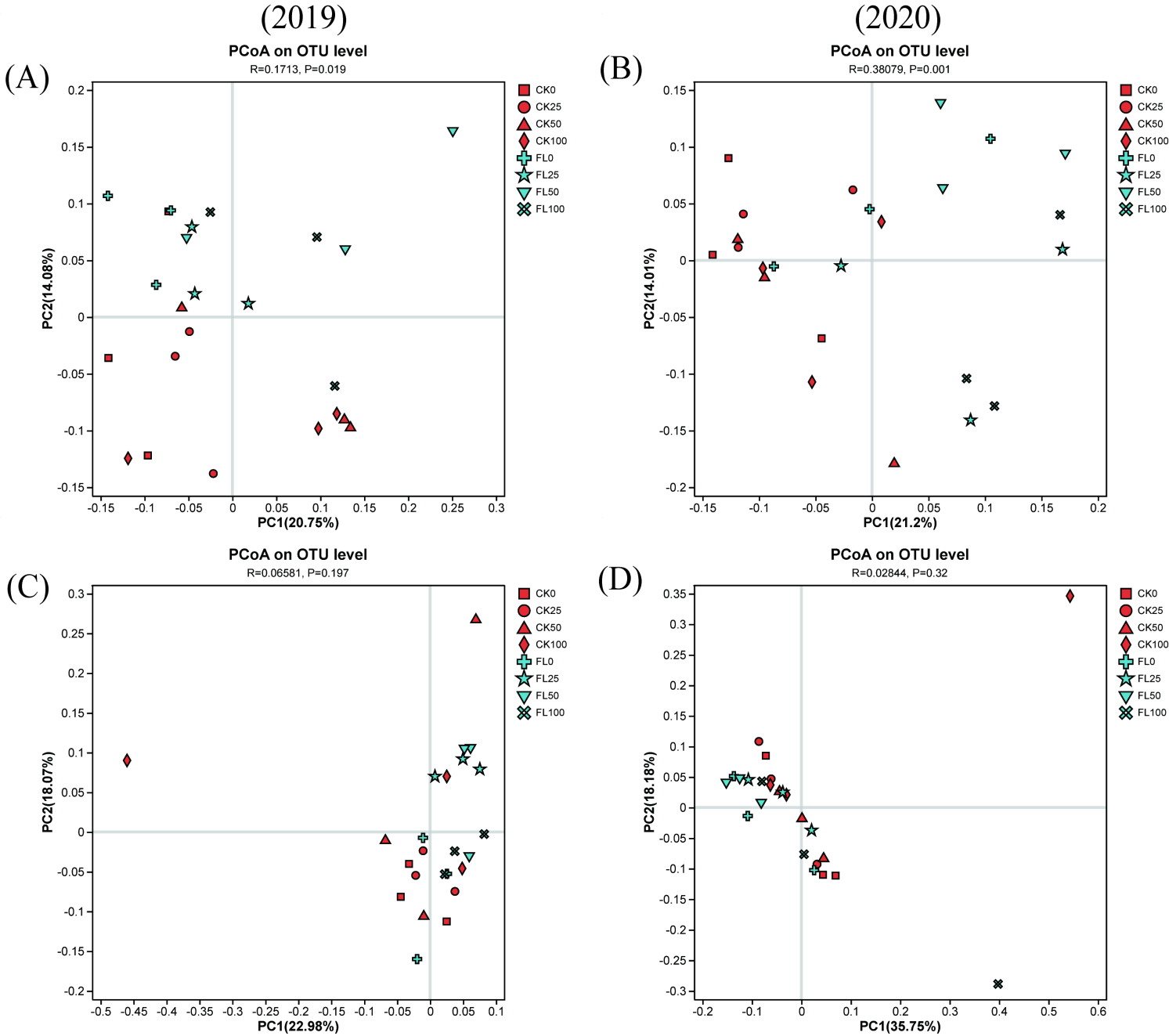


**Fig. S4** Scatter plots of principal coordinates analysis (PCoA) of soil bacterial (A and B) and fungal (C and D) communities based on Bray-Curtis distance.


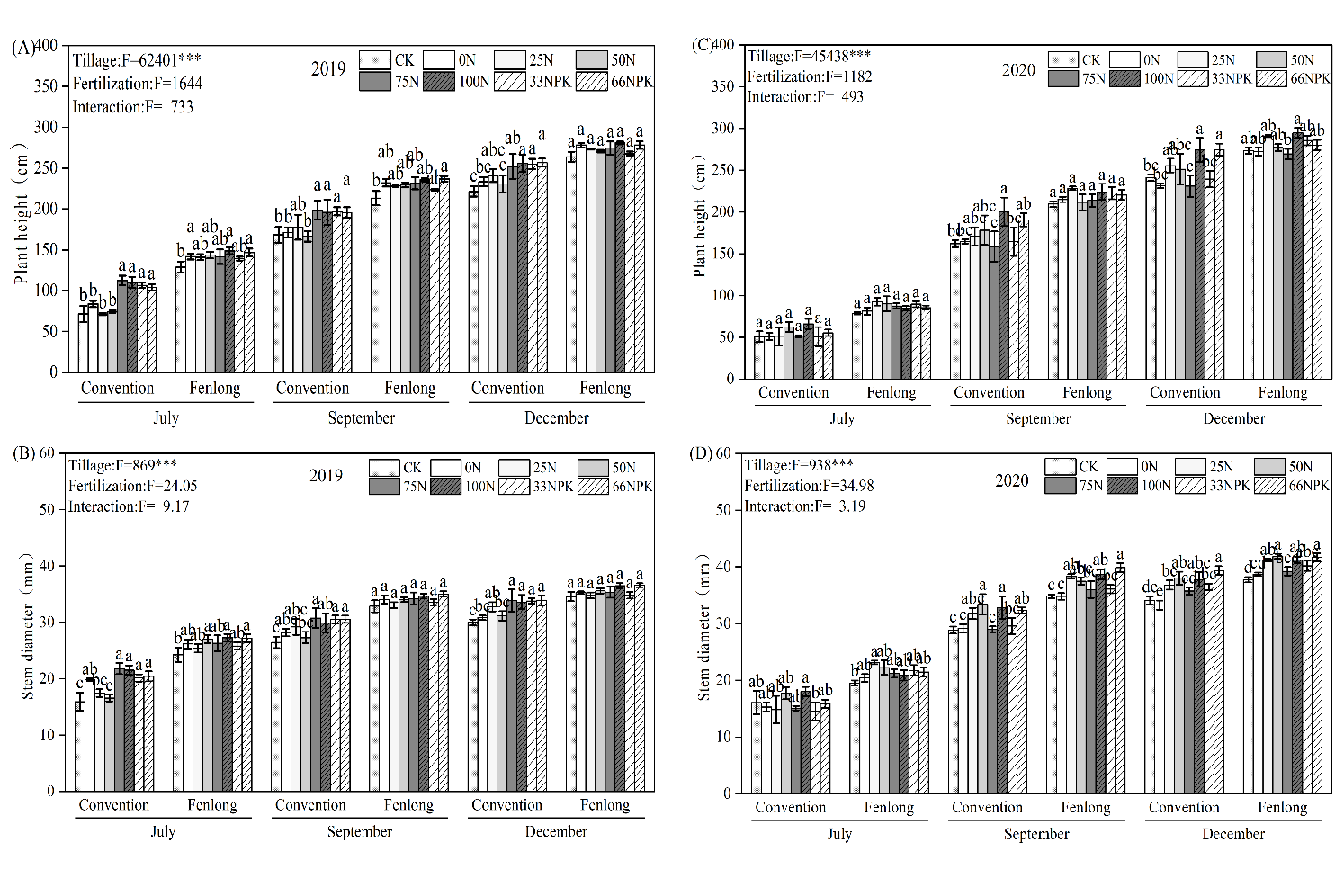


**Fig. S5** Cassava plant height and stem diameter of 2019 (A and B) and 2020 (Cc and D) under different treatments. Different lowercase letters indicate significant differences among treatments at *p* < 0.05. Data are presented as the mean ± standard error (SE). * is significant at the 0.05 level. ** is significant at the 0.01 level. *** is significant at the 0.001 level.


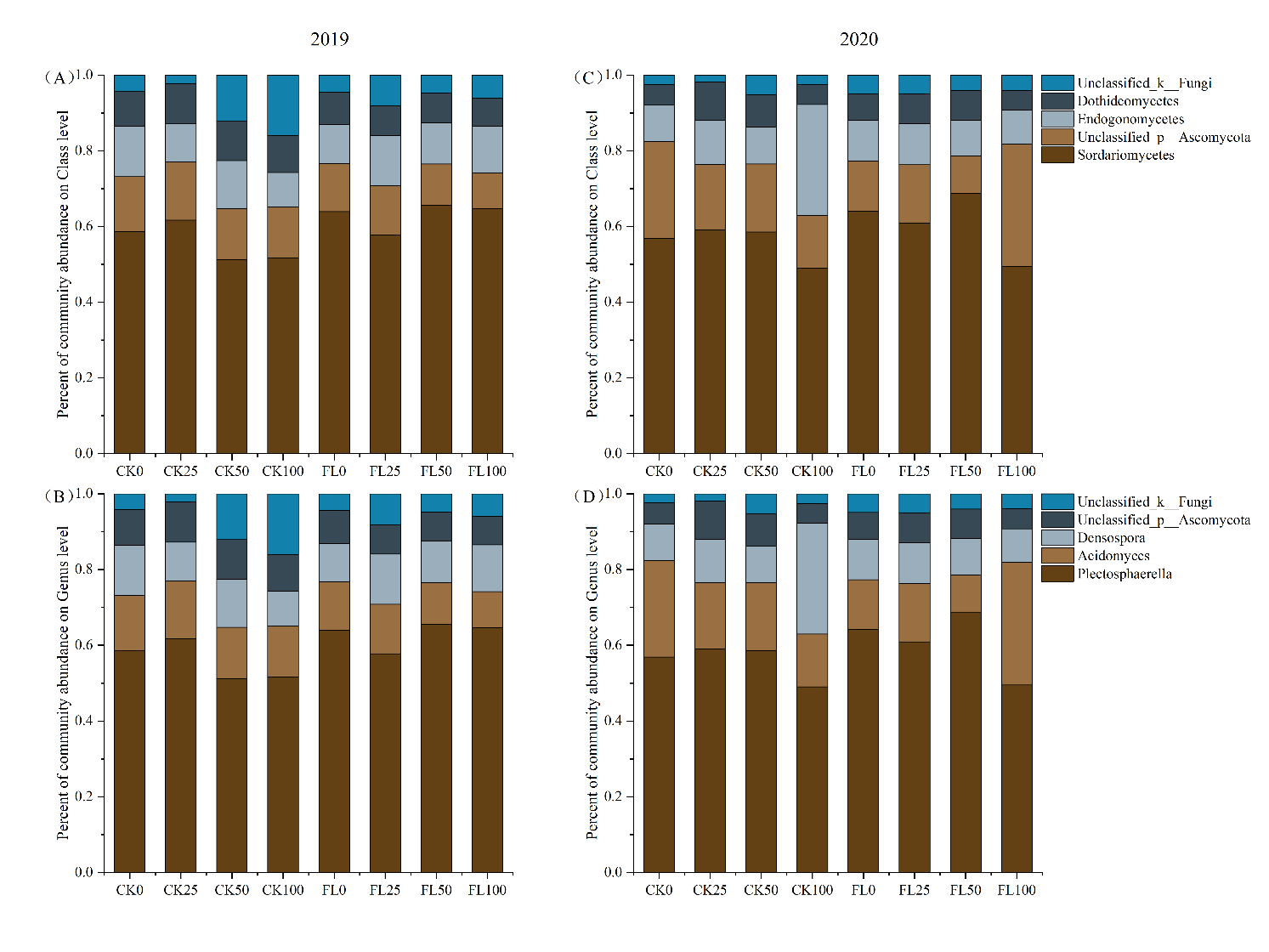


**Fig. S6** Relative abundances (%) of soil fungal community composition at the class and genus levels in 2019 (A and B) and 2020 (C and D).
